# Diffusion segregation and the disproportionate incidence of COVID-19 in African American communities

**DOI:** 10.1098/rsif.2020.0961

**Published:** 2021-01-27

**Authors:** Aleix Bassolas, Sandro Sousa, Vincenzo Nicosia

**Affiliations:** School of Mathematical Sciences, Queen Mary University of London, London E1 4NS, UK

**Keywords:** ethnic segregation, random walks, urban systems, COVID-19, human mobility

## Abstract

One of the most concerning aspects of the ongoing COVID-19 pandemic is that it disproportionately affects people from some specific ethnic and socio-economic minorities. In particular, since from the beginning of the pandemic it has been clear that people from Black and African American backgrounds seem to be hit especially hard by the virus, creating a substantial infection gap. The observed abnormal impact on these ethnic groups could probably be due to the co-occurrence of other known risk factors, including co-morbidity, poverty, level of education, access to healthcare, residential segregation and response to cures, although those factors do not seem able to explain fully and in depth the excess incidence of infections and deaths among African Americans. Here, we introduce the concept of diffusion segregation, that is the extent to which a given group of people is internally clustered or exposed to other groups, as a result of mobility and commuting habits. By analysing census and mobility data on major US cities, we found that the weekly excess COVID-19 incidence and mortality in African American communities at the beginning of the COVID-19 pandemic is significantly associated with their level of diffusion segregation. The results confirm that knowing where people commute to, rather than where they live, is potentially much more important to contain and curb the spreading of infectious diseases.

Ethnic and socio-economic minorities, including African Americans, are quite often subject to considerable disparities connected to health and healthcare, in particular regarding the prevalence of infectious [1–3], chronic [4] and other types of diseases [5,6], and their associated mortality rates. While such disparities can be partially explained by socio-economic indicators, such as access to healthcare services [7], other variables seem to play a relevant role in determining them, including among others residential segregation [8,9]. Due to the particular characteristics of the SARS-CoV-2 virus, which is mainly transmitted through face-to-face contacts [10] and whose bare transmission mechanism does not depend heavily on other pre-existing medical conditions, the substantially higher rates of COVID-19 infections registered among African American communities by different recent studies [11–18] are more than just unusual. Some studies have confirmed that lower income and poorer access to healthcare facilities might have a role in determining an infection gap, but these factors alone seem insufficient to completely explain the observed differences [19–23]. The established connection between health-related disparities and residential segregation points to ethnic segregation, i.e. the tendency of people belonging to the same ethnic group to live closer in space, as a possible additional culprit [24–28]. Indeed, ethnic segregation is a long-standing problem not just across the USA [29], so the idea that the abnormal proportion of COVID-19 infections among African Americans could be due to spatial segregation does not sound unreasonable. However, the results available so far confirm that, although there is a correlation between ethnic segregation and overall incidence of COVID-19 in the population, there seems to be little evidence of an association with infection gap in African Americans [30].

The spread of a non-airborne virus is mostly mediated by direct face-to-face contacts between infected individuals and susceptible ones. This is why the first measures attempting at containing the spread of the SARS-CoV-2 virus [31] focused on the introduction of travel restrictions, social distancing, curfews, and stay-at-home orders [32–35]. In principle, the fact that a certain residential neighbourhood has an overabundance of people belonging to a single ethnic group might have *per se* little or no role in increasing the probability that those people catch COVID-19. Conversely, the fact that a group of people works preferentially in specific sectors, or in specific areas of a city, almost automatically increases the typical number of face-to-face contacts they have during a day, e.g. by forcing them to commute long distances in packed public transport services. Indeed, the distribution of the number of contacts per person is known to be fat-tailed [36], so that most of the infections are actually caused by a relatively small set of individuals, called *super-spreaders* [37,38], who normally have a disproportionately high number of face-to-face contacts. Intuitively enough, super-spreaders are most commonly found among service workers [39]—cashiers, postal workers, clerks, cooks, bus drivers, waiters, etc.—since their job involves being in direct contact with a large number of people on a regular basis. This fact makes super-spreaders more prone to catch diseases that propagate preferentially through direct contacts, like COVID-19 does, and—involuntarily—more efficient at spreading them.

Our hypothesis is that the observed infection gap is most probably due to a prevalence of *super-spreading behaviours* in African American communities, i.e. activities that contribute to increase the typical number and variety of face-to-face contacts of individuals—including for instance their job, habits, social life, commuting and mobility patterns—and that effectively make them more exposed to the infection. In particular, we argue that these super-spreading behaviours are connected to the presence of what we call *diffusion segregation*. By diffusion segregation we mean the extent to which individuals of a certain class or group are either preferentially exposed to other groups, or internally clustered, as a result of their mobility patterns. In this sense, diffusion segregation is somehow complementary to the classical notion of segregation based on residential data, and is instead related to other multi-scalar measures of segregation based on the concept of activity space [40–42]. The interest for the quantification of (ethnic) spatial segregation and its effect on socio-economic inequalities dates back to the 1950s, and has only increased ever since [27,43]. Starting with single scale indicators and pairwise comparisons, the most recent research has moved towards the quantification of segregation at multiple scales, and to the study of the relation between segregation and long-range interactions [41,42,44,45]. Along this line of research, special attention has been devoted to the role of human mobility in urban segregation [44,46], yet most of the works focus on how the effective population of a region changes throughout the day, and not on the actual origin of commuters.

We propose here a method to define and quantify segregation that incorporates information about network topology and commuting patterns, and we show that this diffusion segregation is strongly associated with the abnormal incidence of COVID-19 in African American communities across the USA.

## 1. Results

### 1.1. Model

We propose to quantify the diffusion segregation of a certain group in a urban area in terms of the typical time needed by individuals of that group to get in touch with individuals of other groups when they move around the city. In our model, a city is represented by a graph G where nodes are census tracts and each edge indicates a relation between two areas, namely either physical adjacency or the existence of commuting flows between them. Each node is assigned to a class, according to the ethnicity distribution in the corresponding area (see Methods for details). Then, we consider a random walk on the graph G, and we look at the statistics of class mean first passage times (CMFPT) and class coverage times (CCT). The former is the number of steps needed by a walker starting on a node of a certain class *α* to end up for the first time on a node of class *β*, while the latter is related to the time needed by a random walk to visit all the classes in the system (see Methods for details). The underlying idea is that a random walk through the graph preserves most of the information about correlations and heterogeneity of node classes [47]. Consequently, if a system exhibits diffusion segregation, the statistics of CMFPT and CCT will be substantially different from those observed on a null-model graph having exactly the same set of nodes and edges, but where a node is assigned a class at random from the underlying ethnicity distribution. CMFPT and coverage times of random walks on the adjacency graph of census tracts represent an alternative way of quantifying multi-scalar yet purely residential segregation. By taking into account information about commuting flows among census tracts, this formalism allows one to capture and measure dynamic segregation, i.e. the extent to which people are segregated due to their activity.

We define the normalized CMFPT between class *α* and class *β* as

1.1 $${\tilde{\tau }}_{\alpha \beta }=\frac{\tau_{\alpha \beta }}{\tau_{\alpha \beta }^{\mathrm{null}}},$$

where *τ*_*αβ*_ is the average number of steps needed to reach class *β* when a walker starts from a node of class *α* and $\tau_{\alpha \beta }^{\mathrm{null}}$ is the MFPT from class *α* to class *β* in a null-model graph (see Methods for details). The null-model considered here is the graph with the same topology as the original one, where node classes have been reassigned uniformly at random, i.e. reshuffled by keeping their relative abundance. Note that ${\tilde{\tau }}_{\alpha \beta }$ is a pure number: if ${\tilde{\tau }}_{\alpha \beta }>1$ (resp., ${\tilde{\tau }}_{\alpha \beta }<1$) it means that the expected time to hit a node of class *β* when starting from a node of class *α* is higher (resp., lower) than in the corresponding null-model. In general, a value different from 1 indicates the presence of correlations and heterogeneity in the distribution of classes. Similarly, we define the normalized CCT as

1.2 $${\tilde{\gamma }}_{\alpha }^{\;i}=\frac{\gamma_{\alpha }^{\;i}}{\gamma_{\alpha }^{i,\mathrm{null}}},$$

where $\gamma_{\alpha }^{i}$ is the CCT of a random walk started on node *i* of class *α* and $\gamma_{\alpha }^{i,\mathrm{null}}$ is the corresponding quantity in the null-model (see Methods for details).

**Figure 1.**
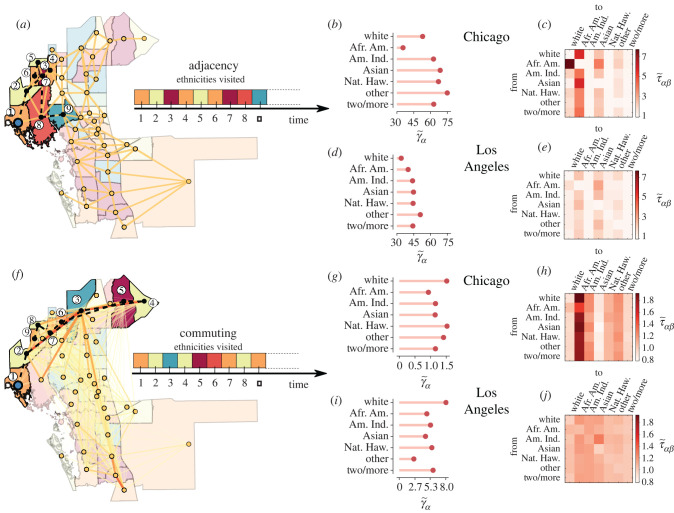
Using typical times of random walks to quantify urban diffusion segregation. The sequence of ethnicities (here indicated by different colours) visited by a random walk over (*a*) the adjacency network or (*f*) the commuting network among census tracts of a city retains relevant information about the presence of spatial correlations in ethnicity distribution. Indeed, the normalized values of class coverage time ${\tilde{\gamma }}_{\alpha }$ (*b,d,g,i*) and class mean first passage time ${\tilde{\tau }}_{\alpha \beta }$ (*c,e,h,j*) of a random walk exhibit different patterns in different cities, and reveal different kinds of ethnic correlations in the adjacency (residential segregation) and in the commuting (dynamic segregation) network of the same city. We show here the values for Chicago or Los Angeles, since Illinois and California have, respectively, one of the highest and one of the lowest COVID-19 incidence gap. Indeed, the residential mean first passage times from African American to White neighbourhoods in the adjacency graph is much higher in Chicago than in Los Angeles, while the dynamic segregation reveals that African Americans are much more exposed to all the other ethnicities in Chicago than in Los Angeles. (Afr. Am.: African Americans; Am. Ind.: American Indians; Nat. Haw.: Native Hawaiian.)

In figure 1, we provide a visual sketch of the model and we show the distributions of CMFPT and CCT in Chicago and Los Angeles. We chose these two specific cities since Illinois and California are two states respectively characterized by a relatively high and a relatively low COVID-19 incidence gap [48,49] (a detail of incidence gap across US states is available in electronic supplementary material, figure S6). Here each node is associated with one of the seven high-level ethnic groups defined by the US Census Bureau [50], with a probability proportional to the abundance of that ethnicity in the corresponding census tract (see Methods for details).

The top panels of figure 1 correspond to the unweighted network A of physical adjacency between census tracts, while the bottom panels are obtained on the weighted network C of typical daily commute flows among the same set of census tracts [51] (see Methods for details). Note that the two graphs have quite different structures: the adjacency graph is planar and each edge connects only nodes that are physically close, while in the commuting graph long edges between physically separated tracts are not only possible, but quite frequent. As a consequence, the adjacency graph provides information about short trips, e.g. for daily shopping and access to local services, while the commuting graph represents long-range trips, e.g. related to commuting to and from work. It is clear that each ethnicity has a peculiar pattern of passage times to the other ethnicities, and this pattern varies across cities. For instance, in Chicago, the two largest values of ${\tilde{\tau }}_{\alpha \beta }$ on the adjacency graph are observed between African Americans and White, and between Asian and African Americans. Conversely, in Los Angeles the two largest values of ${\tilde{\tau }}_{\alpha \beta }$ are between African American and Asian and between other and Asian. As expected, the profile of ${\tilde{\tau }}_{\alpha \beta }$ for a given class is quite different if we consider the commuting network instead of the adjacency graph. In Chicago, the largest value of ${\tilde{\tau }}_{\alpha \beta }$ is from White to African American, while in Los Angeles there are a lot of pairs of classes with pretty similar values of ${\tilde{\tau }}_{\alpha \beta }$, indicating that in this city diffusion segregation for African Americans is less prominent than in Chicago. The value of ${\tilde{\gamma }}_{\alpha }$ for African Americans is especially low in Chicago, but noticeably different from that of the other ethnicities in Los Angeles. As we shall see in a moment, ${\tilde{\gamma }}_{\alpha }$ is related to the isolation of a class, so that lower values correspond to increased exposure to all the other classes.

**Figure 2.**
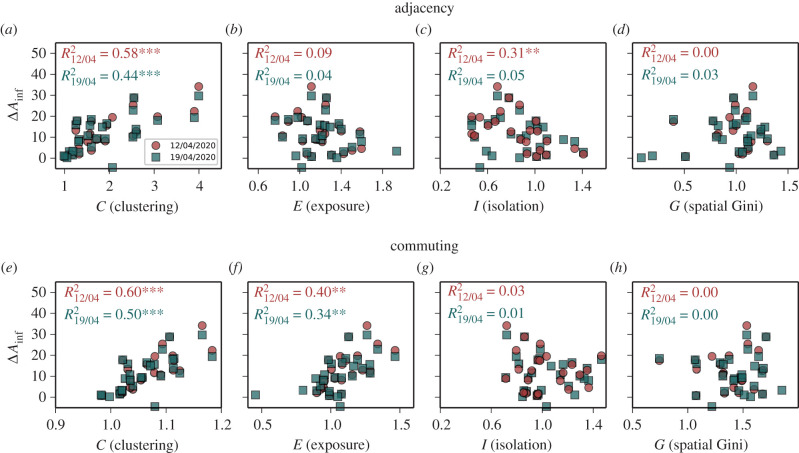
Correlation between incidence gap and diffusion segregation in the early stages of the epidemics. The incidence gap Δ*A*_inf_ across US states in the first two weeks after extensive lock-down measures were enforced exhibits somehow strong correlation with the residential segregation based on CMFPT and CCT on the adjacency (*a–d*) and the dynamic one (*e–h*). In particular, the diffusion clustering *C* (*a,e*) is always positively correlated with Δ*A*_inf_, the diffusion exposure *E* (*b,f*) is positively correlated with Δ*A*_inf_ only in the commuting network, and the diffusion isolation *I* (*c,g*) is negatively associated with incidence gap only in the adjacency network. Note that classical measures of residential segregation, like the spatial Gini coefficient (*d,h*), are instead poorly or not correlated at all with incidence gap. Each colour corresponds to a temporal snapshot of the dataset, red for 12 April 2020 and blue for 19 April 2020. (*: *p* < 0.05, **: *p* < 0.01, ***: *p* < 0.001.)

### 1.2. Measuring diffusion segregation

We constructed three indices to quantify the diffusion segregation of African Americans in a given city based on the values of CMFPT and CCT, which respectively capture the clustering (*C*), exposure (*E*) and isolation (*I*) of these communities. All the indices are based on the ratio of CMFPT and CCT between African Americans and other ethnicities, and are computed on the adjacency and on the commuting graph. In the following, the subscript *A* will always indicate African Americans, while the subscript *O* will indicate all the other ethnicities. We start by defining the following quantities:

$$\begin{array}{rl} {\bar{\tau }}_{AA} & =\langle {\tilde{\tau }}_{AA}\rangle \\ {\bar{\tau }}_{AO} & =\frac{\sum_{\alpha \neq A}M^{\alpha }\langle {\tilde{\tau }}_{A\alpha }\rangle }{\sum_{\alpha \neq A}M^{\alpha }}\\ {\bar{\tau }}_{OA} & =\frac{\sum_{\alpha \neq A}M^{\alpha }\langle {\tilde{\;\tau }}_{\alpha A}\rangle }{\sum_{\alpha \neq A}M^{\alpha }}\\ \mathrm{and}\;\;\;{\bar{\tau }}_{OO} & =\frac{\sum_{\alpha ,\beta \neq A}\langle {\tilde{\tau }}_{\alpha \beta }\rangle M^{\alpha }M^{\beta }}{\sum_{\alpha ,\beta \neq A}M^{\alpha }M^{\beta }}, \end{array}$$

where *M*^*α*^ is the total number of individuals of class *α* present in a city. In practice, ${\bar{\tau }}_{AA}$ is the normalized CMFPT from African Americans to African Americans; ${\bar{\tau }}_{AO}$ is the normalized CMFPT from African Americans to all the other classes (weighted by ethnicity distribution); ${\bar{\tau }}_{OA}$ is the normalized CMFPT from all the other classes to African Americans (again, weighted by ethnicity distribution); and ${\bar{\tau }}_{OO}$ is the normalized CMFPT among all the other ethnicities. We define the diffusion clustering of African Americans in a city as

1.3 $$C=\frac{{\bar{\tau }}_{AO}}{{\bar{\tau }}_{OO}}$$

so that values of *C* larger than 1 indicate that a walker started at an African American neighbourhood will need more time to hit neighbourhoods of any other ethnicity than a walker started at any other neighbourhood. Similarly, we define the diffusion exposure of African Americans to other ethnicities as

1.4 $$E=\frac{{\bar{\tau }}_{OA}}{{\bar{\tau }}_{AO}},$$

where values of *E* larger than 1 indicate that it is easier for a walker started at an African American neighbourhood to visit any other ethnicity than vice versa. Finally, we define the isolation of African Americans neighbourhoods as

1.5 $$I=\frac{1}{N}\sum_{i=1}^{N}\frac{{\tilde{\gamma }}_{A}^{i}}{{\tilde{\gamma }}_{O}^{i}},$$

where ${\tilde{\gamma }}_{A}^{i}$ is the normalized CCT from African American neighbourhoods and ${\tilde{\gamma }}_{O}^{i}$ is the average of the normalized CCT for neighbourhoods of other ethnicities. Here *N* is the number of nodes in the graph. Note that values of *I* larger than 1 indicate that the normalized CCT from nodes of class *A* (African American) is higher than the CCT from nodes of all the other classes. The state-level value of each index is obtained as an average of the corresponding index on the cities of the state, weighted by the population of each city.

### 1.3. Diffusion segregation and infection gap

In order to study the relation between diffusion segregation and COVID-19 infection gap, we considered two temporal datasets of weekly percentage of African Americans infected by and deceased due to COVID-19 for each state in the USA [48,49], and adjacency and commuting graphs constructed from census tract information on more than 130 US cities (see Methods for more details and electronic supplementary material, table S1, for the complete list of cities). We calculated the incidence gap Δ*A*_inf_ in each state as the difference between the percentage of infected of that state that are African Americans and the percentage of African American population in the same state. Hence, positive values of Δ*A*_inf_ correspond to a disproportionate incidence of COVID-19 in African American communities.

In figure 2, we show the scatter plots of the average diffusion clustering, exposure, and isolation of African Americans at state level, and of the corresponding COVID-19 infection gap in the first two weeks after major lock-down measures were introduced across the USA. We chose these two temporal snapshots because the number of confirmed infected individuals in a week actually depends on their contacts up to two weeks before, due to the COVID-19 incubation period [52]. The top panels report the residential segregation of census tracts, while the bottom panels correspond to dynamic segregation. Interestingly, there exists a quite strong correlation between diffusion segregation and the disproportionate number of infected in African American communities. In particular, the diffusion clustering of African Americans in a state correlates positively and quite strongly with the infection gap observed in that state in the first two weeks of the dataset, both on the adjacency (respectively, *R*^2^ = 0.58 and *R*^2^ = 0.44 in the first two weeks) and in the commuting network (respectively *R*^2^ = 0.60 and *R*^2^ = 0.50). This means that if African American citizens normally require more time than citizens from other ethnic groups before ending up in a non-African American neighbourhood, then the incidence gap will be considerably higher.

The role of diffusion exposure is even more interesting. Indeed, the residential exposure on the adjacency network is not correlated at all with incidence gap, while the dynamic one is a good predictor of incidence gap (respectively, *R*^2^ = 0.40 and *R*^2^ = 0.36). Conversely, the diffusion isolation of African Americans in the adjacency graph is negatively correlated with incidence gap in the early stages of the epidemics (*R*^2^ = 0.31). Similar results are obtained when we consider the correlation with the death gap Δ*A*_dec_ (see electronic supplementary material, S1 and figure S2). In particular, diffusion isolation exhibits a somehow stronger correlation with death gap (*R*^2^ = 0.27). It is worth noting that ethnic segregation based on residential data, e.g. as measured by the spatial Gini coefficient, has no significant correlation with incidence gap, as well as the diffusion segregation of other ethnicities (see electronic supplementary material, figure S3). Conversely, other related measures of segregation based on CMFPT or on activity space exhibit intermediate values of correlation, but still lower than the diffusion segregation measures proposed above (see electronic supplementary material, S3 and figures S9 and S10).

In table 1, we report a summary of the correlations between infection gap and a variety of classical and more recent spatial segregation measures, as measured in the adjacency and the commuting graphs (see Methods for a description of the measures considered in the table). Interestingly, most of those classical measures do not show any significant correlation with infection gap, with the only exception of the Perimeter/Area Ratio Spatial Dissimilarity and those based on [55], which attain correlations close to those obtained with the diffusion segregation when measured on the commuting graph. In addition, both the distance decay isolation and exposure display a slight correlation for a few dates. See electronic supplementary material, table S2, for the correlations with death gap.

**Table 1.** Table of correlations (Pearson *R*^2^) obtained with additional widely used segregation indicators. All the indices are obtained by comparing (ratio) the segregation of African Americans with the average of the other ethnicities. Except for *C*^*f*^, *E*^*f*^, ${\bar{\sigma }}_{g}$ and spatial Gini in the commute graph, all indicators were calculated using the PySAL package in Python [53].

date	network	12/04	15/04	19/04	22/04	26/04	29/04	03/05	06/05	10/05	13/05	17/05
Bound. Spat. Dissim. [53]	adj.	0.04	0.00	0.01	0.03	0.01	0.00	0.00	0.00	0.00	0.00	0.00
Bound. Spat. Dissim. [53]	com.	0.07	0.02	0.01	0.03	0.02	0.00	0.00	0.00	0.00	0.00	0.00
Spat. Dissim. [53]	adj.	0.06	0.16*	0.07	0.04	0.11	0.16*	0.19**	0.13*	0.09	0.08	0.11*
Spat. Dissim. [53]	com.	0.00	0.05	0.02	0.02	0.08	0.10	0.11	0.09	0.05	0.05	0.08
Per./area ratio Spat. Diss. [53]	adj.	0.19*	0.08	0.11	0.14*	0.14*	0.06	0.04	0.08	0.04	0.04	0.03
Per./area ratio Spat. Diss. [53]	com.	0.20*	0.34**	0.27**	0.32***	0.48***	0.46***	0.36***	0.40***	0.27**	0.27***	0.30***
Dist. Decay Exposure [53]	adj.	0.12	0.21*	0.09	0.10	0.23**	0.19**	0.16*	0.21**	0.07	0.06	0.12*
Dist. Decay Exposure [53]	com.	0.07	0.16*	0.08	0.10	0.22**	0.19*	0.16*	0.20**	0.07	0.07	0.13*
Dist. Decay Isolation [53]	adj.	0.17*	0.28**	0.13	0.14*	0.31***	0.26**	0.21**	0.25**	0.11*	0.09	0.14*
Dist. Decay Isolation [53]	com.	0.10	0.21*	0.10	0.13*	0.29**	0.25**	0.20**	0.24**	0.10	0.09	0.14*
${\bar{\sigma }}_{g}$ [54]	adj.	0.02	0.00	0.06	0.09	0.04	0.01	0.01	0.02	0.02	0.02	0.01
${\bar{\sigma }}_{g}$ [54]	com.	0.19*	0.09	0.03	0.01	0.02	0.02	0.01	0.01	0.00	0.00	0.00
*E*^*f*^ [55]	adj.	0.36**	0.45***	0.26**	0.24**	0.40***	0.35***	0.19**	0.18**	0.09	0.07	0.09
*E*^*f*^ [55]	com.	0.16	0.07	0.02	0.01	0.03	0.02	0.01	0.01	0.00	0.00	0.00
*C*^*f*^ [55]	adj.	0.19*	0.32**	0.19*	0.15*	0.23**	0.23**	0.16*	0.11*	0.05	0.05	0.08
*C*^*f*^ [55]	com.	0.47***	0.43***	0.32**	0.22**	0.26**	0.27**	0.11	0.09	0.06	0.04	0.05
spatial Gini [53]	adj.	0.00	0.00	0.03	0.04	0.06	0.01	0.01	0.02	0.02	0.02	0.01
spatial Gini [53]	com.	0.00	0.01	0.00	0.00	0.00	0.02	0.03	0.02	0.00	0.00	0.02
Moran’s I [54]	adj.	0.00	0.01	0.00	0.00	0.00	0.02	0.03	0.02	0.00	0.00	0.02
Moran’s I [54]	com.	0.00	0.01	0.00	0.00	0.00	0.02	0.03	0.02	0.00	0.00	0.02

**Figure 3.**
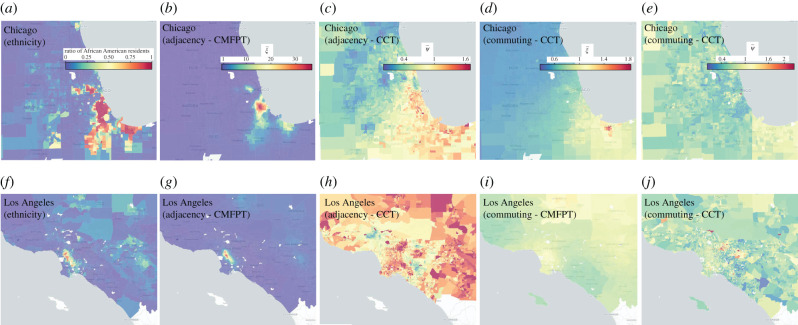
Distribution of local diffusion segregation. The distribution of the fraction of African American population living in each census tract (*a,f*) is mostly unrelated to the local clustering index ${\tilde{\xi }}_{i}$ (*b,d,g,i*) and to the local isolation index ${\tilde{\psi }}_{i}$ (*c,e,h,j*). The figure shows the result for Chicago (top panels) and for Los Angeles (bottom panels). Overall, there is little correlation between the density of African American residents and the diffusion segregation of African Americans in an area. This explains why diffusion segregation indices in a city correlate quite strongly with the COVID-19 infection gap, while no strong association with residential segregation has been found so far. In fact, the correlation between diffusion segregation and the infection gap would only be possible if it provides a different information from the ratio of African American population.

The improved predictive power of diffusion segregation with respect to the traditional residential approach can be better explained by looking at how residential data and diffusion segregation are distributed across a city. In figure 3 we show the heat-maps of abundance of African American residents in Chicago and Los Angeles together with the local segregation indices ${\tilde{\xi }}_{i}$ and ${\tilde{\psi }}_{i}$, respectively, related to diffusion clustering and to diffusion isolation, both residential and dynamic (see the definitions provided in Methods; additional maps for Detroit and Houston are reported in electronic supplementary material, S2 figure S4). It is true that in Chicago ${\tilde{\xi }}_{i}$ in the adjacency graph is still somehow correlated with the fraction of African American population (see electronic supplementary material, figure S5). But the distribution of ${\tilde{\xi }}_{i}$ in the commuting graph is totally different. In particular, the regions characterized by residential clusters of African Americans exhibit lower values of ${\tilde{\xi }}_{i}$, meaning that the commuting patterns make those neighbourhoods overall less isolated. Conversely, new hot-spots are identified in the South-Eastern region of Gary, likely due to the fact that people in this region do not commute much to the city centre anyway. Similarly, the areas of Los Angeles with the largest local isolation are not the neighbourhoods with a higher percentage of African Americans residents, rather the suburbs characterized by high commuting.

### 1.4. Combined effects of diffusion segregation and use of public transport

**Figure 4.**
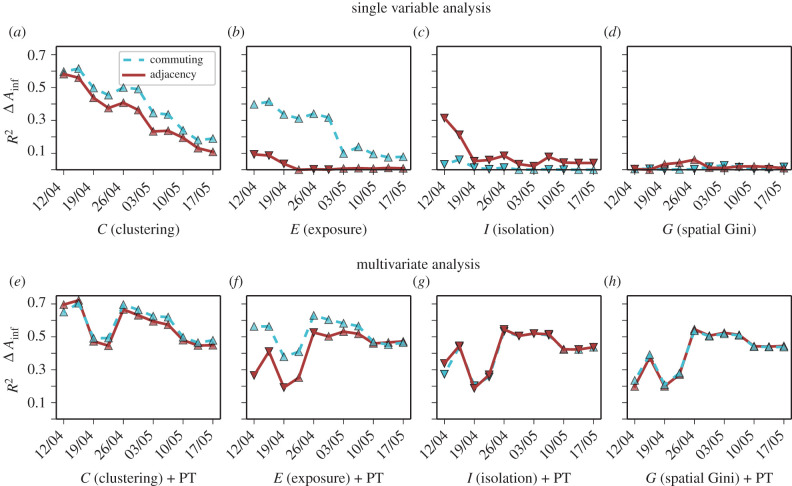
Temporal evolution of incidence gap correlations and multivariate analysis with public transport usage. Evolution of the Pearson correlation (*R*^2^) between African American incidence gap and (*a*) diffusion clustering, (*b*) diffusion exposure, (*c*) diffusion isolation, (*d*) spatial Gini coefficient on the adjacency (solid red lines) and commuting graphs (dashed blue lines). Multivariate analysis of the same indices and usage of public transportation by African Americans for (*e*) diffusion clustering, (*f*) diffusion exposure, (*g*) diffusion isolation, (*h*) spatial Gini coefficient. The type of marker indicates the sign of the correlation (triangles pointing up for positive correlations, and down for negative correlation). Given the uneven temporal reporting of ethnicity data, each temporal snapshot has a slightly different number of US states. We have also tried alternative formulations for *C* and *E* obtaining significant correlations, as shown in electronic supplementary material, figure S9. The multivariate analysis was performed using R and the ANOVA model type II in the car package.

In figure 4*a*–*d,* we show the correlation between infection gap and diffusion segregation measures as the pandemic progresses. Unsurprisingly, the correlation with any single measure decreases over time for all the indices, and both on the adjacency and on the commuting graphs. Similar results are found for the correlation with death gap in African Americans (see electronic supplementary material, figure S7) as well as with a second dataset we had access to [49] (see electronic supplementary material, figure S8). The main reason for the observed decreases is that once large-scale mobility restrictions are put in place—as happened between the end of March and the beginning of April 2020 across all the US states with stay-at-home orders and curfews—the overall mobility structure of each city is massively disrupted. As a result, super-spreading behaviours due to usual commuting patterns are substantially reduced, and the contagion progresses mainly through face-to-face interactions happening close to the residential place of each individual, which are not captured well by CMFPT and CCT on the commuting graph.

The importance of local transport after lock-downs are enforced is evident in figure 4*e*–*h*, where we show the results of the multivariate analysis of the same set of segregation indices shown in figure 2 and of the fraction of African American population using public transport in each city (see Methods for details). The combination of diffusion segregation and the use of public transport correlates quite consistently with the incidence gap. These findings are made more relevant by the fact that the incidence gap in African Americans in the same period is quite poorly correlated with the overall usage of public transport in the population, as well as with a variety of other socio-economic indices, as shown in electronic supplementary material, figure S11. Since cities are complex interconnected systems, it is plausible to hypothesize that segregation and public transport usage are related in subtle and intricate ways, so that it is practically impossible to establish whether the former has caused the latter, or instead the two phenomena have coevolved over time.

### 1.5. Combined effects of diffusion segregation and socio-economic status

**Figure 5.**
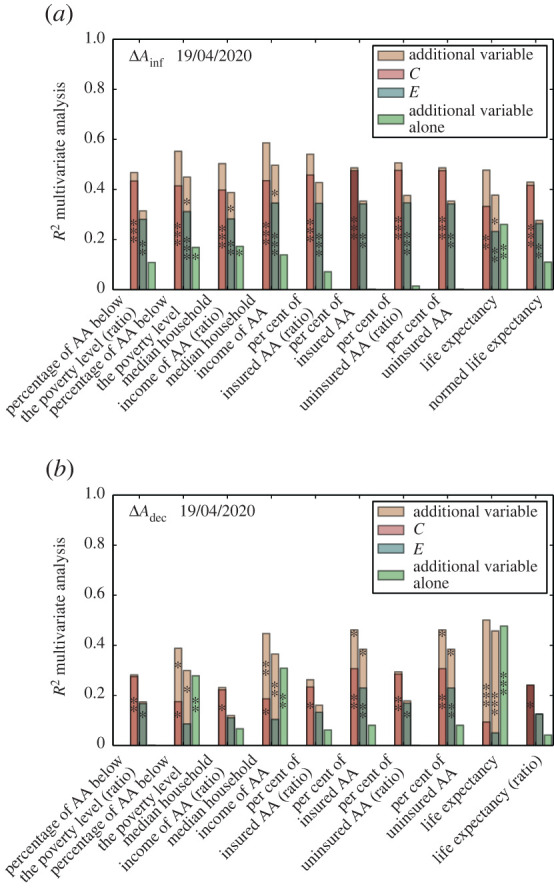
Multivariate analysis with socio-economic indicators. Correlation (*R*^2^) between (*a*) incidence gap and (*b*) death gap obtained by a multivariate analysis that includes either diffusion clustering *C* or diffusion exposure *E* together with one of the socio-economic indicators we have considered. In light green we show the correlation with each of the indicators alone, in dark green the correlation obtained for *E* in the multivariate analysis, in red the correlation obtained for *C* in the multivariate analysis and in yellow the correlation obtained with the socio-economic indicator for each of the segregation indicators. Diffusion segregation is more strongly associated with the measured infection gap, while socio-economic variables are more correlated with death gap.

It is important to note, however, that other socio-economic indicators, such as level of access to healthcare through insurance, income, or life expectancy can potentially aggravate the impact of infectious diseases on African American communities. For this reason, we have performed an extensive multivariate analysis by using dynamic clustering and exposure together with each of those indicators. The results by 19 April 2020 are reported in figure 5 for incidence gap (figure 5*a*) and death gap (figure 5*b*). See electronic supplementary material, figures S12 and S13, for the results by 12 April 2020. We note that dynamic segregation measures have more explanatory power for the incidence gap than any of the other socio-economic indicators alone. In particular, the percentage of insured and uninsured African Americans seems to have little to no influence on the infection gap. Conversely, life expectancy, income level, and percentage of uninsured African Americans in the population exhibit a very good correlation with death gap. This is somehow expected, since life expectancy and access to healthcare can in principle have an impact on increasing the mortality risk of a disease (e.g. due to the fact that critical COVID-19 patients often develop acute pneumonia and cannot survive without the support of a mechanical ventilator), but in general these factors do not play a significant role in increasing the infection risk in a particular group. It is worth noting that median household income, but in particular life expectancy, also show some significative correlation with infection gap, despite their explanatory power being much smaller than that of diffusion segregation indices. This is most probably due to the fact that income level is a quite strong indication of job type, with essential workers normally found at the bottom of the salary scale. Similarly, a lower life expectancy level, which is in general due to a mixture of other variables and might be related to a variety of other underlying health conditions, can favour the development of the clinical complications typically associated with COVID-19. Interestingly, almost all the structural socio-economic variables considered actually add a bit of useful information to the overall picture, as the correlation with incidence gap in the multivariate case is always slightly larger than those obtained with dynamic segregation indicators alone. But still, their association with incidence gap is rather small. These results are in agreement with previous works performed in both the USA [11,12,14] and the UK [19], in which disparities in infection and death rates remained substantial even after removing confounding factors such as comorbidity or socio-economic status.

## 2. Discussion

The vulnerability and the socio-economic disparities that characterize African American communities have been a prominent issue in the USA long before the current COVID-19 pandemic, including strong disparities in their mortality rates [6]. Despite such disparities having also been observed in other infectious diseases such as HIV/AIDS [2] or in the vaccination against influenza [5], the discovery of a COVID-19 incidence gap in Black and African American population is somehow unexpected, since no specific biological risk factor has been strongly associated with an increased vulnerability to the virus of any specific ethnic group, the mechanism of transmission is different from other viruses and no vaccine is yet available. Hence, the most unbiased assumption to explain such a disproportionate incidence, which in some areas is three to five times higher than the fraction of African American population, is that it should be related to behavioural, social and historical factors, rather than to biological ones. The most frequently whispered theory is that African Americans are more exposed to COVID-19 because they are more frequently employed in service works. This explanation is quite reasonable, since service workers normally have hundreds of face-to-face interactions during a day. Indeed, some recent studies have estimated that the switching to remote-working was mainly available to people employed in non-essential services, and amounted to 22–25% of the work force before April 2020 [56]. As expected, service workers are one of those categories for which the option to switching to remote-working during the lock-down was not available at all, especially in sectors deemed vital for the functioning of a country during lock-downs, including food production and retailers, healthcare, transportation and logistics. According to the US Labour Force Statistics [57], the occupations with the highest concentration of African Americans are indeed jobs characterized by face-to-face interaction, and most of them fall in the area of *essential* jobs: postal service sorters/processors (42%), nursing (37%), postal service clerks (35%), protective service workers (34%) and barbers (32%). It would not then come as a surprise to discover that one of the major early COVID-19 outbreaks happened in South Dakota, in a meat-processing plant, whose workers were mainly of African American background [58].

The potential relation between ethnicity and mobility was somehow hinted at in a recent study [59] which found that the decrease in the usage of subway transport in New York during the lock-down was uneven across ethnicities, with African Americans experiencing the smallest relative drop. But unfortunately, the publicly available data about COVID-19 incidence do not contain detailed-enough information about socio-economic characteristics of infected individuals, so drawing an association between African Americans, employment in essential service jobs, availability of remote-working options and increased COVID-19 exposure is very hard.

An interesting finding of the present work is that the combination of diffusion segregation and use of public transport seems to explain the persistence of infection gap throughout the early phases of the pandemic (when pre-existing social determinants have been removed). Indeed, before lock-downs are put in place, African Americans are found to be more exposed to the virus, mainly due to the structure of their daily commuting patterns. After lock-downs are enforced, instead, they are more likely to pass the virus over to other African Americans, as a result of the high levels of clustering and isolation of these communities measured in the adjacency graphs of census tracts, which are a more reliable proxy for face-to-face interactions when long-distance commuting is disrupted. In general, the states where African Americans are more exposed with respect to long-distance trips are also those where they are more clustered with respect to short-range mobility (the rank correlation between the two measures is 0.62, as shown in electronic supplementary material, figure S1).

The importance of considering the interaction of different classes due to urban mobility has recently received some attention [40,41,44,46,54,55,60–62]. The methodology presented here brings together the long-lasting dichotomy between segregation and exposure [41,42,44,45] allowing for the quantification of both and, more interestingly, showing that segregation and exposure can appear at the same time in certain contexts. The method based on the passage times of random walkers that we have presented here can be considered a generalization of multi-scale and k-nearest neighbour approaches [41,42,45], which by definition can be applied to any type of network, either based on mobility or on any other subject such as transportation infrastructures. The results presented here also raise several other interesting questions, especially regarding the interplay between the segregation measured on the adjacency graph and the commuting graph of a urban system, or whether any attempt to reduce the diffusion segregation of certain ethnic groups could potentially improve the livability of a city. While our analysis has focused in the home–work mobility due to the wider availability of such information, random walkers can be used to evaluate the role played by any kind of urban mobility, including pedestrian flows and mobility connected to leisure and entertainment.

In this sense, it is quite interesting that the simple diffusion model we used here to quantify the presence of residential and dynamic segregation, and the corresponding indices of clustering, exposure, and isolation, are able to unveil a relatively strong correlation between the structure of mobility in a metropolitan area and the excess incidence of COVID-19 infections and deaths in African Americans. Although the model we consider uses relatively small and coarse-grained information about a city—placement of census tracts, local ethnicity distribution, and commuting trips among them—the strong correlation between diffusion segregation and incidence gap allows one to draw a simple conclusion: when it comes to predicting the exposure of a group to a non-airborne virus, knowing the places where the members of that group commute for work is more important and more relevant than knowing where they actually live. This is also confirmed by the quite poor association of incidence gap with other classical and more recent measures of racial segregation (see electronic supplementary material, S3 and figure S10).

We cannot conclude that the existence and magnitude of the COVID-19 infection gap among African Americans is entirely due to the diffusion segregation of those communities, since many other factors could play a role in the dynamics of infection propagation. But we can definitely affirm that, among the variety of different socio-economic factors that might have an impact on infection gap, diffusion segregation and usage of public transportation play a very significant role. On the one hand, the results presented in this work suggest that there is a strong connection between the place where people live and the way they move around the city, so that policy makers should definitely take into account diffusion segregation when modelling the spread of a disease in an urban area, and in predicting the impact of specific countermeasures. In particular, an effective way to mitigate incidence consists in reducing as much as possible long-distance trips for people that are naturally more exposed to face-to-face contacts, e.g. due to their occupation. On the other hand, the fact that several structural socio-economic indicators, including life expectancy and income level, have a strong association with death gap suggests that the problem is much more complicated to tackle. Any effective and successful approach to minimize the casualties due to a pandemic needs to strike a quite delicate balance between immediate tactical measures, aiming at reducing the ongoing spread of the disease among those groups that are more exposed to it, and long-term strategical measures, aiming at reducing the causes of abnormal infection and death prevalence in specific ethnic and socio-economic minorities.

## 3. Methods

### 3.1. Geographical network datasets

Ethnicity data were obtained from [50] and include the data from the 2010 US decennial census. Commuting trips data come from the 2011 US census [51], focusing on the seven highest-level ethnicity classes, namely: White, Black or African American, American Indian and Alaska Native, Asian, Native Hawaiian and Other Pacific Islander, Some Other Race, Two or More Races. Population is updated to the latest American Community Survey 2014–2018 5-year Data Release [63].

For each metropolitan area, we constructed two distinct spatial networks. The first one is the *adjacency network*, denoted by A and obtained by associating each cell to a node and connecting two nodes with a link if the corresponding cells border each other. Note that A is an undirected and unweighted graph. The second graph is the *commuting network*, denoted as C. In this network, each node is a tract and the directed and weighted link *ω*_*ij*_ between node *i* and *j* indicates the number of commuting trips from *i* to *j* as obtained from census information. To reconstruct a mobility network that resembles the real one (which amounts to something between 30% and 40% of the total mobility in a city) we aggregated both the trips from home to work and the corresponding return trip from work to home.

Each node of the adjacency network A preserves information about the ethnicity distribution on the corresponding census tract. We use the *N* × *Γ* matrix $M=\{m_{i,\alpha }\}$, where *Γ* is the number of ethnicities present in the city and *N* is the number of areal units. The generic element *m*_*i*,*α*_ of M indicates the number of citizens of ethnicity *α* living on node *i*. We denote by *M*_*i*_ = {*m*_*i*,*α*_} the vector of population distribution at node *i*, and by $M^{\alpha }=\sum_{i=1}^{N}m_{i,\alpha }$ the total number of individuals of class *α* present in the system. In the commuting network C, instead, we attribute to each node *i* both the resident population at the corresponding tract and the population commuting to node *i*, so that the abundance of individuals of class *α* on node *i* becomes

3.1 $${\tilde{m}}_{i,\alpha }=m_{i,\alpha }+\sum \omega_{\;ji}m_{\;j,\alpha },$$

where *ω*_*ji*_ is the number of daily commuting trips from node *j* to node *i*. By doing so we aim to capture the fact that a commuter to cell *i* will potentially have face-to-face interactions with both residents in that area and other workers commuting to that area every day. Moreover, since the commuting network C accounts for both work–home and home–work trips, the adjusted population on the commuting network accounts for the potential contacts that individuals had at the origin of a trip as well.

### 3.2. Class mean first passage time

Let us consider a generic graph G(V,E) with |E|=K edges on |V|=N nodes, and a colouring function f : V→χ that assigns to each node *i* of G a discrete label *f*_*i*_ from the finite set *χ* with cardinality |*χ*| = *Γ*. Let us also consider a random walk on G, defined by the transition matrix Π = {*π*_*ij*_} where *π*_*ji*_ is the probability that the walk jumps from node *i* to node *j* in one step. On the adjacency network A, we use a uniform random walk, i.e. $\pi_{\;ji}=\frac{1}{k_{i}}$, while on the commuting graph C, we have $\pi_{\;ji}=\frac{\omega_{ij}}{s_{i}}$, where $s_{i}=\sum_{\;j}\omega_{ij}$ is the out-strength of node *i*.

Here, we focus on the statistical properties of the trajectories $W_{i}=\{f_{i_{0}},f_{i_{1}},\ldots \}$ of node labels visited by the random walk *W* at each time when starting from *i*_0_ = *i* at time *t* = 0. This dynamics contains information about the existence of correlation and heterogeneity in the distribution of colours. For instance, if the graph *G* is a regular lattice and the function *f* associates colours to nodes uniformly at random, we expect that, for long-enough time, all the trajectories starting from each of the *N* nodes will be statistically indistinguishable.

We denote as *T*_*i*,*α*_ the MFPT from a given node *i* to nodes of class *α*, i.e. the expected number of steps needed for a walk starting on *i* to visit for the first time any node *j* such that *f*_*j*_ = *α*. We can write a self-consistent forward equation for *T*_*i*,*α*_ [64]:

3.2 $$T_{i,\alpha }=1+\sum_{\;j=1}^{N}(1-\delta_{\;f_{\;j},\alpha })\pi_{\;ji}T_{\;j,\alpha }.$$

The MFPT *τ*_*βα*_ from class *α* to class *β* is defined as

3.3 $$\tau_{\alpha \beta }=\frac{1}{N_{\alpha }}\sum_{\;j=1}^{N}T_{\;j,\beta }\delta_{\;f_{\;j},\alpha },$$

where *N*_*α*_ is the number of nodes in the graph associated with class *α*. Note that in practice the value of *τ*_*αβ*_ is obtained as an average over many realizations of the random walk.

A notable issue of the MFPT defined in equation (3.3) is the fact that its values might depend on the specific distribution of colours (i.e. on their abundance) and on the size of the network under consideration, which makes it difficult to compare MFPT computed on different systems. To obviate this problem, we define the normalized CMFPT between class *α* and class *β* as

3.4 $${\tilde{\tau }}_{\alpha \beta }=\frac{\tau_{\alpha \beta }}{\tau_{\alpha \beta }^{\mathrm{null}}},$$

where $\tau_{\alpha \beta }^{\mathrm{null}}$ is the MFPT from class *α* to class *β* obtained in a null-model graph. The null-model considered here is the graph having the same topology as the original one, and where node colours have been reassigned uniformly at random, i.e. reshuffled by keeping their relative abundance.

### 3.3. Class coverage time

The coverage time is classically defined as the number of steps needed by a random walk to visit a certain percentage of the nodes of a graph when starting from a given node *i* [64]. In the case of a network with coloured nodes, a walk started at node *i* will be associated with the generic trajectory $W_{i}=\{f_{i_{0}},f_{i_{1}},f_{i_{2}},\ldots \}$ of node labels visited by the walk at each time. Since we are interested in quantifying the heterogeneity of ethnicity distributions, we consider the time series $W_{i}=\{M_{i},M_{i_{1}},M_{i_{2}},\ldots \},$ where $M_{i_{t}}=\{m_{i_{t},\alpha }\}$ is the distribution of ethnicities at node *i*_*t*_ visited by the walk at time *t*. If we consider the trajectory up to time *t*, the vector $Q_{i_{t}}=\frac{1}{H_{t}}\sum_{\tau }M_{i_{\tau }}$ is the distribution of ethnicities visited up to time *t* by the walker started at *i* (here *H*_*t*_ is a normalization constant that guarantees $\sum_{\;j}{\left\{Q_{i_{t}}\right\}}_{\;j}=1$). We quantify the discrepancy between $Q_{i_{t}}$ and the global ethnicity distribution across the city $P=(1/H^{\prime })M^{⊺}1_{N}$ by means of the Jensen–Shannon divergence

3.5 $$J(P∥Q_{i_{t}})=\frac{1}{2}[D(P∥\mu )+D(Q_{i_{t}}∥\mu )],$$

where $\mu =\frac{1}{2}(P+Q_{i_{t}})$ and *D*(*P*∥ *Q*) is the Kullback–Liebler divergence between *P* and *Q*. We define the CCT from node *i* at threshold *ɛ* as

3.6 $$\gamma^{i}=\underset{t}{\mathrm{argmin}}\{J(P∥Q_{i_{t}})\leq \varepsilon \}$$

and the associated normalized CCT

3.7 $${\tilde{\gamma }}^{i}=\frac{\gamma^{i}}{\gamma^{i,\mathrm{null}}},$$

where *γ*^*i*,null^ is the CCT from node *i* in a null-model where the colours associated with the nodes have been reshuffled uniformly at random.

### 3.4. CMFPT and CCT in census networks

In the case of ethnicity distributions in geographical networks, each node is not uniquely associated with a colour, but it has instead a local distribution of ethnicities. Nevertheless, the formalism for the computation of CMFPT and CCT described above can still be used in this case as well. We consider a stochastic colouring function $\tilde{\;f}\;:\;V\to X$ that associates to each node *i* of the adjacency graph one of the *Γ* = 7 ethnicities *α* with probability $m_{i,\alpha }/\sum_{\beta }m_{i,\beta }$ (respectively, with probability ${\tilde{m}}_{i,\alpha }/\sum_{\beta }{\tilde{m}}_{i,\beta }$ in the commuting graph), i.e. proportionally to the abundance of ethnicity *α* in node *i*.

To compute the CMFPT we consider *S* independent realizations of the stochastic colouring process for each network. On each realization ℓ, we estimate the MFPT among all classes as in equation (3.3), and the corresponding null-model MFPT. Then, we compute the average CMFPT from class *α* to class *β* as

3.8 $$\langle {\tilde{\tau }}_{\alpha \beta }\rangle =\frac{\sum_{\ell =1}^{S}\tau_{\alpha \beta }^{\left(\ell \right)}}{\sum_{\ell =1}^{S}{\left(\tau_{\alpha \beta }^{\mathrm{null}}\right)}^{\left(\ell \right)}},$$

where $\tau_{\alpha \beta }^{\left(\ell \right)}$ is the CMFPT computed on the ℓ-th realization and ${\left(\tau_{\alpha \beta }^{\mathrm{null}}\right)}^{\left(\ell \right)}$ is the corresponding value in the null-model. For each system, we computed $\tau_{\alpha \beta }^{\mathrm{null}}$ on 500 realizations of the null model, with 500 independent colour assignments per realization, and 2000 walks per node.

The computation of CCT works in a similar way. In order to take into account the heterogeneous distribution of ethnicities across nodes, before a walker starts from node *i* we sample one of the ethnicities present on *i*, according to their local abundance at *i* {*m*_*i*,*β*_}, and we attribute node *i* to it. Then, we compute the CCT from node *i* of class *α* as the average CCT from node *i* across all the walks starting from *i* where node *i* was actually assigned to class *α*, and we call this quantity $\gamma_{\alpha }^{i}$. Note that in this case, we consider the trajectories $W_{i}^{\alpha }=\{M_{i}^{\alpha },M_{i_{1}}^{\alpha },M_{i_{2}}^{\alpha },\ldots \}$ where $M_{i_{\ell }}^{\alpha }$ is the distribution of ethnicities at the ℓ-th node visited by the walker, which does not include class *α*. The class *α* is also removed from the global distribution P when computing the Jensen–Shannon divergence so that it concentrates on the average time taken by an individual belonging to class *α* to meet all the other classes. The normalized CCT from class *α* when starting from node *i* is defined as

3.9 $${\tilde{\gamma }}_{\alpha }^{i}=\frac{\gamma_{\alpha }^{i}}{\gamma_{\alpha }^{i,\mathrm{null}}},$$

where $\gamma_{\alpha }^{i,\mathrm{null}}$ is the CCT from node *i* of class *α* in the null-model. Finally, the average CCT from class *α* is simply obtained as

3.10 $${\tilde{\gamma }}_{\alpha }=\frac{1}{N}\sum_{i=1}^{N}{\tilde{\gamma }}_{\alpha }^{i}.$$

For all the computations of CCT shown in the paper we considered averages over 5000 walks per node and we set *ɛ* = 0.0018. This value was obtained by using the Pinsker inequality for the Kullback–Leibler divergence and imposing a total variation distance smaller than 6%. It corresponds to the precision level at which the proportion of classes observed by the walker compares to the city level, while obtaining values smaller than 6% is computationally expensive and requires on average significantly more steps.

### 3.5. Local indices of diffusion ethnic segregation

We define two local segregation indices for African Americans in a census tract *i*. The first index is based on CMFPT

3.11 $${\tilde{\xi }}_{i}=\frac{\sum_{\alpha \neq A}{\tilde{T}}_{i,\alpha }}{(\Gamma -1){\tilde{T}}_{i,A}},$$

where ${\tilde{T}}_{i,\alpha }$ corresponds to the normalized MFPT to a generic class *α* when a random walker starts from node *i*, while ${\tilde{T}}_{i,A}$ is the CMFPT to African Americans tracts. Values of ${\tilde{\xi }}_{i}$ larger than 1 indicate that the time to reach any other ethnicity is higher than the time needed to reach African Americans, hence indicating a local clustering of African Americans around node *i*.

The local index of isolation is derived from CCT

3.12 $${\tilde{\psi }}_{i}=\frac{{\tilde{\gamma }}_{A}^{i}}{{\tilde{\gamma }}_{O}^{i}},$$

where ${\tilde{\gamma }}_{A}^{i}$ is the CCT from node *i* for African Americans and ${\tilde{\gamma }}_{O}^{i}$ is the average CCT from node *i* for all the other ethnicities. In general, if ${\tilde{\psi }}_{i}$ is larger than 1 then African Americans living at node *i* are isolated, since they will require more time to visit all the other classes than required by individuals from other ethnicities.

### 3.6. Classical measures of spatial segregation

The set of measures of spatial segregation reported in table 1 include:

— The classical Spatial Dissimilarity index [65] which can be interpreted as a measure of how different the social composition of neighbourhoods is, on average, from the social composition of the study area.
— Two variations of spatial dissimilarity index, the Boundary and Perimeter/Area ratio [66], which take into account the length of the common boundary between two areal units and their shapes.
— The Distance Decay Exposure and the Distance Decay Isolation indices proposed in [67]. The former indicates the probability that an individual belonging to a group meets anywhere in space someone from other groups while the latter accounts for the probability of meeting someone from the same group. We used default values of *α* and *β* parameters for the Isolation and Exposure measures, 0.6 and 0.5, respectively, which aims to estimate the extent of the proximity within the same unit.
— The spatial Gini index, that infers the contribution of spatial neighbouring pairs to overall inequality across a set of regions. We considered the share of inequality in non-neighbour components to obtain the correlations [28] which can be written as
  3.13 $$SG=\frac{\sum_{i=1}^{N}\sum_{\;j=1}^{N}(1-w_{ij})|x_{i}-x_{\;j}|}{2N^{2}<x>}.$$
  However, this formulation is designed for binary networks, which is the case of the adjacency graph but not of the commuting network. To take into account that those are weighted network, we have modified it as
  3.14 $$SG_{w}=\frac{\sum_{i=1}^{N}\sum_{\;j=1}^{N}(1-w_{ij}/w_{\max})|x_{i}-x_{\;j}|}{2N^{2}<x>}.$$
— The Moran’s I Global Auto-correlation, which measures spatial auto-correlation based on both feature locations and feature values simultaneously [27].

The indices calculated on the adjacency network considered row standardization of the spatial weights matrices which were based on binary associations, i.e. 1 for neighbouring areas and 0 otherwise. For the commuting network, weights were kept in their original form as they represent asymmetric passenger flows. The measures were computed using the PySAL package [28]. For all measures, we obtained the value for each of the Γ classes and computed the ratio of the corresponding value for African Americans to the average of the other classes, so that the comparison with the diffusion segregation is meaningful.

## Supplementary Material

Supplementary material to Diffusion Segregation and the disproportionate incicence of COVID-19 in African American communities

## Data accessibility

The data related to the percentage of infected African Americans were obtained from two different sources, namely https://blackdemographics.com/black-covid-19-tracker and https://covidtracking.com/race. The first dataset reports the number of infected and deceased of each ethnicity along with those unknown. To calculate the percentage of African Americans, we have removed first the unknown from the total, otherwise our analysis would also capture the fraction of unknown. For the other dataset, we just extract the data they provide in tables. The public transportation dataset was obtained from the 2018 American Community Survey from US Census Bureau [50]. It includes information about the percentage of public transportation usage per ethnicity and state. All the processed datasets and networks are available at https://datadryad.org/stash/dataset/doi:10.5061/dryad.hqbzkh1f9.
