# Supplementary material for: Diffusion segregation and the disproportionate incidence of COVID-19 in African American communities
Source: J R Soc Interface. 2021 Jan 27;18(174):20200961. doi: 10.1098/rsif.2020.0961 (PMC7879774; doi:10.1098/rsif.2020.0961)
Supplement: Supplementary material to Diffusion Segregation and the disproportionate incicence of COVID-19 in African American communities [file rsif20200961supp1.pdf]

Supplementary Material
Diffusion segregation and the disproportionate incidence of COVID-19 in African American communities
Journal of the Royal Society Interface

Aleix Bassolas, Sandro Sousa, Vincenzo Nicosia

List of Figures

S1 Kendall tau τ_k correlation between each of the four indices studied in the main manuscript computed either over the adjacency or the commuting graphs. 3
S2 Relation between the difference on the percentage of deceased African American as a function of the four indices considered 4
S3 Isolation index of all ethnicities as a function of the infected rate gap of COVID-19 cases in the African American population on the adjacency and commuting network. 5
S4 Maps of local segregation in American Cities 6
S5 Pearson correlation coefficient between the each of the local metrics of segregation $\widetilde{\xi}$ and $\widetilde{\psi}$ and the local ratio of African American population 7
S6 The temporal evolution of the difference in percentage of infected and deceased African Americans by state. 8
S7 Evolution of the Pearson and Spearman correlation (R^2) found between the difference in percentage of deceased African Americans and the four indices studied in the main manuscript 9
S8 Evolution of the Pearson and Spearman correlation (R^2) found between the difference on the infected African Americans and the four indices studied in the main manuscript using another data source 10
S9 Evolution of the Pearson and Spearman correlation (R^2) found between the difference on the percentage of infected African Americans and the alternative indices proposed C' and E' 11
S10 Correlations between the incidence of COVID-19 in African American population and traditional the segregation indices 12
S11 Correlations between the incidence of COVID-19 in African American population and a set of socio-economic and mobility indicators 15
S12 Multivariate analysis for the incidence of COVID-19 in African American population as a function of a set of socio-economic indicators and the clustering (G) and exposure (E) by the 12th of April 2020 16
S13 Multivariate analysis for the deceased by COVID-19 in African American population as a function of a set of socio-economic indicators and the clustering (G) and exposure (E) by the 12th of April 2020 16

List of Tables

S1 Table of cities studied 2
S2 Table of correlations for the death gap of African Americans (Pearson R^2) obtained with additional widely used segregation indicators. 13

City name	City name	City name	City name
Albany-Schenectady	Albuquerque-Santa Fe-Las Vegas	Appleton-Oshkosh-Neenah	Asheville-Brevard
Atlanta–Athens-Clarke County–Sandy Springs	Bend-Redmond-Prineville	Birmingham-Hoover-Talladega	Bloomington-Bedford
Bloomington-Pontiac	Bloomsburg-Berwick-Sunbury	Boston-Worcester-Providence	Bowling Green-Glasgow
Brownsville-Harlingen-Raymondville	Buffalo-Cheektowaga	Cape Coral-Fort Myers-Naples	Cape Girardeau-Sikeston
Charleston-Huntington-Ashland	Charlotte-Concord	Chattanooga-Cleveland-Dalton	Chicago-Naperville
Cincinnati-Wilmington-Maysville	Cleveland-Akron-Canton	Clovis-Portales	Columbia-Moberly-Mexico
Columbia-Orangeburg-Newberry	Columbus-Auburn-Opelika	Columbus-Marion-Zanesville	Columbus-West Point
Corpus Christi-Kingsville-Alice	Dallas-Fort Worth	Davenport-Moline	Dayton-Springfield-Sidney
Denver-Aurora	DeRidder-Fort Polk South	Des Moines-Ames-West Des Moines	Detroit-Warren-Ann Arbor
Dixon-Sterling	Dothan-Enterprise-Ozark	Eau Claire-Menomonie	Edwards-Glenwood Springs
Elmira-Corning	El Paso-Las Cruces	Erie-Meadville	Fargo-Wahpeton
Fayetteville-Lumberton-Laurinburg	Findlay-Tiffin	Fort Wayne-Huntington-Auburn	Fresno-Madera
Gainesville-Lake City	Grand Rapids-Wyoming-Muskegon	Green Bay-Shawano	Greensboro–Winston-Salem–High Point
Greenville-Spartanburg-Anderson	Greenville-Washington	Harrisburg-York-Lebanon	Harrisonburg-Staunton-Waynesboro
Hartford-West Hartford	Hickory-Lenoir	Hot Springs-Malvern	Houston-The Woodlands
Huntsville-Decatur-Albertville	Idaho Falls-Rexburg-Blackfoot	Indianapolis-Carmel-Muncie	Ithaca-Cortland
Jackson-Brownsville	Jackson-Vicksburg-Brookhaven	Jacksonville-St. Marys-Palatka	Johnson City-Kingsport-Bristol
Johnstown-Somerset	Jonesboro-Paragould	Joplin-Miami	Kalamazoo-Battle Creek-Portage
Kansas City-Overland Park-Kansas City	Knoxville-Morristown-Sevierville	Kokomo-Peru	Lafayette-Opelousas-Morgan City
Lafayette-West Lafayette-Frankfort	Lake Charles-Jennings	Lansing-East Lansing-Owosso	Las Vegas-Henderson
Lexington-Fayette–Richmond–Frankfort	Lima-Van Wert-Celina	Lincoln-Beatrice	Little Rock-North Little Rock
Longview-Marshall	Los Angeles-Long Beach	Louisville/Jefferson County–Elizabethtown–Madison	Lubbock-Levelland
Macon-Bibb County–Warner Robins	Madison-Janesville-Beloit	Manhattan-Junction City	Mankato-New Ulm-North Mankato
Mansfield-Ashland-Bucyrus	Martin-Union City	McAllen-Edinburg	Medford-Grants Pass
Memphis-Forrest City	Miami-Fort Lauderdale-Port St. Lucie	Midland-Odessa	Milwaukee-Racine-Waukesha
Minneapolis-St. Paul	Mobile-Daphne-Fairhope	Modesto-Merced	Monroe-Ruston-Bastrop
Morgantown-Fairmont	Moses Lake-Othello	Mount Pleasant-Alma	Myrtle Beach-Conway
Nashville-Davidson–Murfreesboro	New Bern-Morehead City	New Orleans-Metairie-Hammond	New York-Newark
North Port-Sarasota	Oklahoma City-Shawnee	Omaha-Council Bluffs-Fremont	Orlando-Deltona-Daytona Beach
Oskaloosa-Pella	Paducah-Mayfield	Parkersburg-Marietta-Vienna	Pensacola-Ferry Pass
Peoria-Canton	Philadelphia-Reading-Camden	Pittsburgh-New Castle-Weirton	Portland-Lewiston-South Portland
Portland-Vancouver-Salem	Pueblo-Canyon City	Pullman-Moscow	Quincy-Hannibal
Raleigh-Durham-Chapel Hill	Rapid City-Spearfish	Redding-Red Bluff	Reno-Carson City-Fernley
Richmond-Connersville	Rochester-Austin	Rochester-Batavia-Seneca Falls	Rockford-Freeport-Rochelle
Rocky Mount-Wilson-Roanoke Rapids	Rome-Summerville	Sacramento-Roseville	Saginaw-Midland-Bay City
St. Louis-St. Charles-Farmington	Salt Lake City-Provo-Orem	San Jose-San Francisco-Oakland	Savannah-Hinesville-Statesboro
Seattle-Tacoma	Sioux City-Vermillion	South Bend-Elkhart-Mishawaka	Spokane-Spokane Valley-Coeur d'Alene
Springfield-Branson	Springfield-Greenfield Town	Springfield-Jacksonville-Lincoln	State College-DuBois
Steamboat Springs-Craig	Syracuse-Auburn	Tallahassee-Bainbridge	Toledo-Port Clinton
Tucson-Nogales	Tulsa-Muskogee-Bartlesville	Tyler-Jacksonville	Victoria-Port Lavaca
Virginia Beach-Norfolk	Visalia-Porterville-Hanford	Washington-Baltimore-Arlington	Wausau-Stevens Point-Wisconsin Rapids
Wichita-Arkansas City-Winfield	Williamsport-Lock Haven	Youngstown-Warren	

Table S1: Table of cities studied

Section S1: Correlations between the incidence of COVID-19 among the African American population cases and ethnic segregation

Comparison between metrics

We detail the cities studied in Table S1, it is important to note that those are the cities studied disregarding if those states provide ethnic information on the impact of COVID-19.

To evaluate how similar are the rankings of each of the metrics studied in the main manuscript we have calculated the Kendall τ_k between each pair of rankings. Fig. S1 displays the values of τ_k between each pair of the four metrics studied in the main manuscript computed in the adjacency and commuting graphs. For instance, there is a high correlation between the index C computed in the adjacency and the commuting graphs while for the exposure index E there is almost no correlation between both. Likely pointing out that exposure can only be effectively measured by including the commuting network. Another additional observation is the connection between C and E measured on the commuting graph, which seems to point out that in those states where African Americans are more segregated they are also more exposed.

Correlations between the segregation indices and other measures of COVID-19 incidence

The COVID-19 data used was obtained from [1] and includes several temporal snapshots until mid-may. The main variables we used are the difference on infected/deceased African Americans, where 0 would mean that the percentage of African Americans in the population of a state is the same than the percentage of infected.

We have also evaluated how our metrics relate to the difference in percentage among the deceased African Americans (See Fig. S2). Despite many more factors such as the age or underlying health conditions might influence the deceased individuals, still, most of the correlations remain significant

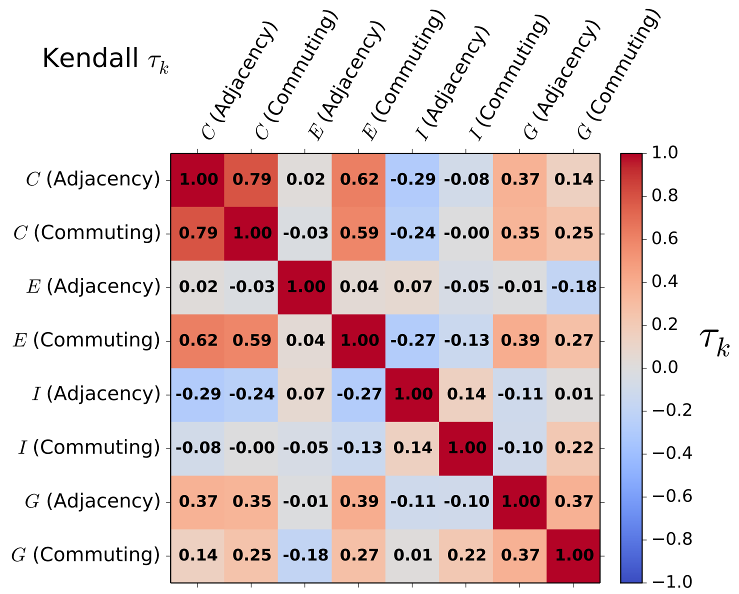

Fig. S1: Kendall tau τ_k correlation between each of the four indices studied in the main manuscript computed either over the adjacency or the commuting graphs.

to some extent, especially those related to their exposure. Moreover, those indices computed on the commuting network seem to be more informative than those based on the adjacency, which seems to point out that residential segregation provides only a partial picture of ethnic inequality. Mobility is also crucial to understand the mixing between different ethnicities, it is not only relevant where certain ethnicities live but also where they work and with whom they interact when they do so.

Correlations with other isolation indices

Ideally, each ethnicity α should be compared with the corresponding ratio to the overall population and the incidence of COVID-19 cases. As this data was not available during the preparation of this work, we look at the gap ΔA_{inf} of African American and the relation with the Isolation level of all other ethnicities in this study. Considering the quantity for all other ethnicities defined as:

$$\tilde{\gamma}_O^i = \frac{1}{\Gamma - 1} \sum_{\beta \neq \alpha} \tilde{\gamma}_\beta^i \tag{1}$$

the Isolation index for an ethnicity α is given by:

$$I_\alpha = \frac{1}{N} \sum_{i=1}^{N} \frac{\tilde{\gamma}_\alpha^i}{\tilde{\gamma}_O^i} \tag{2}$$

Correlations with the infection rate gap ΔA_{inf} on the adjacency and commute network are reported on Fig. S3 for all ethnicities. Quantities are obtained from the COVID-19 data set at 2 different periods, 12-04-2020 and 19-04-2020 respectively. The corresponding R^2 of the Pearson correlation is reported in the inset of each panel. There is a negative correlation in **B** which indicates that less isolated African Americans have a higher incidence of infection cases. Whites **A** and Native Hawaiians **E** exhibit no correlation while the remaining ethnicities **C-D** and **F-G** have a positive R^2 which decreases over time. We found no correlation for any ethnicity on the commuting network.

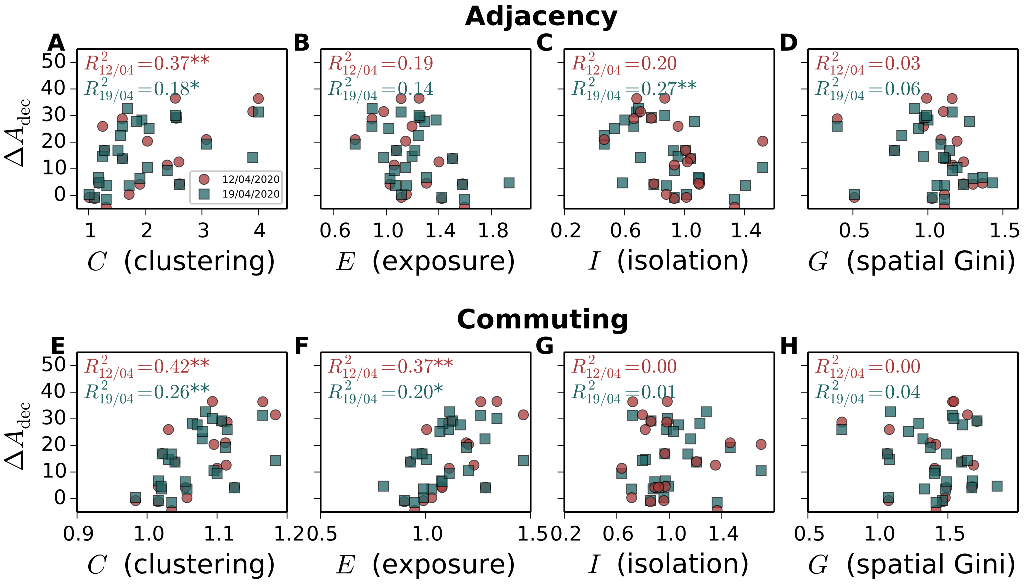

Fig. S2: **Relation between the difference on the percentage of deceased African American as a function of the four indices considered. A-D** Indices computed over the adjacency network: **A** C (clustering), **B** E (exposure), **C** I (isolation), **D** G (spatial Gini). **E-H** Indices computed over the commuting network: **E** C (clustering), **F** E (exposure), **G** I (isolation), **H** G (spatial Gini). Each of the colours corresponds to a temporal snapshot of the data set, red for 12/04/2020 and blue for 19/04/2020. The R^2 is computed as the square of the linear correlation coefficient.

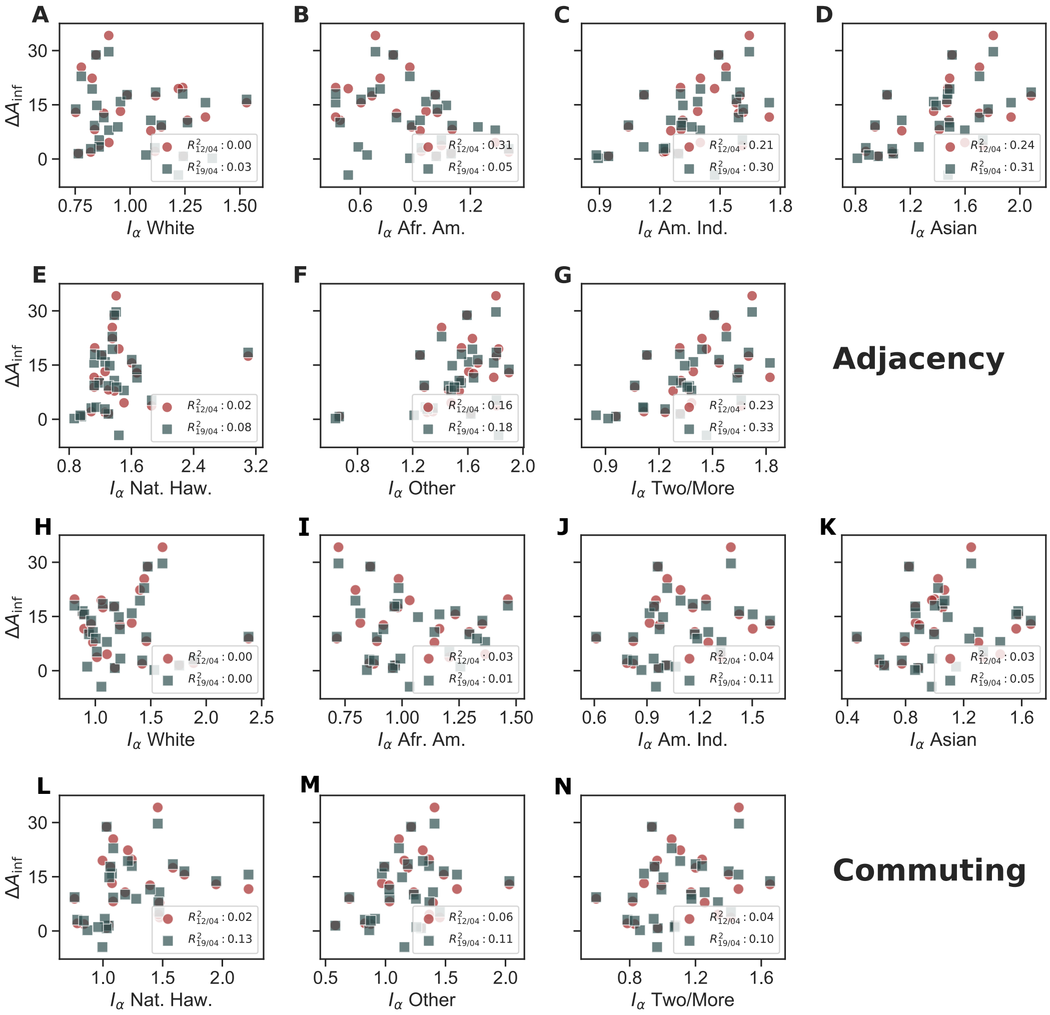

Fig. S3: Isolation index of all ethnicities as a function of the infected rate gap of COVID-19 cases in the African American population on the adjacency and commuting network network. African American is the only ethnicity to exhibit a negative correlation of isolation and ΔA_{inf}, suggesting that a higher infection rate can be related to lower isolation.

Section S2: Local segregation maps through CMFPT and CCT and spatial correlation

In the main manuscript, we show the values for the local segregation indices ξ andψ for Chicago and Los Angeles showing that there were significant differences on their spatial distribution as well as in their maximum values. Here we provide also results for Detroit and Houston to show that again there are significant differences. In this case, Detroit is the most populated city in Michigan, which is one of the states with highest values in most of the indices considered and Houston is the most populated city in Texas, which is a state with consistent low values in most segregation indices. Regarding the impact of COVID-19 among the African Americans of those states, in Michigan the gap is around 34% in early April and 24% in mid-may. In Texas, instead, the gap is around 2% at the beginning of April and 5% in mid-May.

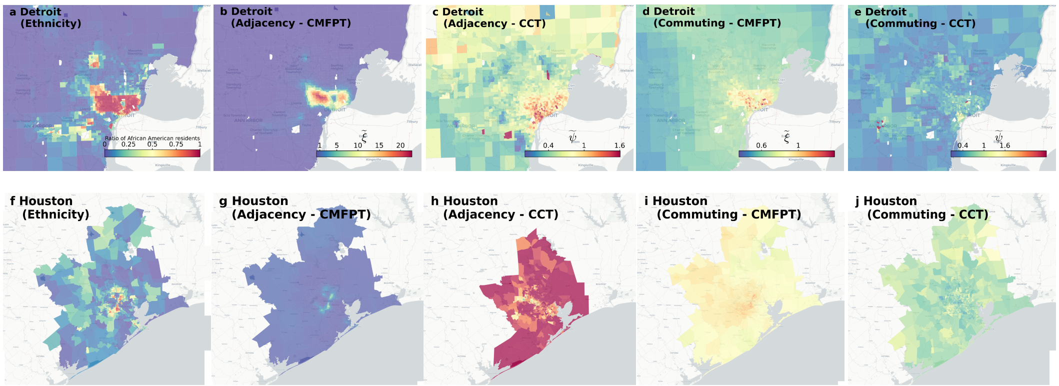

Fig. S4: **Maps of local segregation in American cities.** Ratio of African American population and local segregation indices computed with CMFPT and CCT in **a-e** Detroit and **f-j** Houston. For Detroit: **a** Ratio of African American population, **b-c** $\widetilde{\xi}$ and $\widetilde{\psi}$ computed over the adjacency graph and **d-e** $\widetilde{\xi}$ and $\widetilde{\psi}$ computed over the commuting graph. For Houston: **f** Ratio of African American population, **g-h** $\widetilde{\xi}$ and $\widetilde{\psi}$ computed over the adjacency graph and **i-j** $\widetilde{\xi}$ and $\widetilde{\psi}$ computed over the commuting graph.

In the main manuscript and Fig. S4 we plot the local measures of segregation in each of the census tracts of Chicago, Los Angeles, Detroit and Houston. Those maps display certain common patterns that we quantify in Fig. S5. Therein we have calculated the Kendall τ_k correlation coefficient performing pairwise comparisons of the values for each tract unit. Additionally to the segregation indices, we also compared the values for the ratio of African American population. It is relevant to note that while the value of τ_k for the ratio of African American population and $\widetilde{\xi}$ computed in the adjacency graph is around 0.8 for all the four cities studied, there are stronger variations when $\widetilde{\xi}$ is computed in the commuting graph – i.e., 0.81 in Detroit and 0.55 in Houston – meaning that the effect of commuting in the segregation of African American population can display strong differences across cities and, therefore, mobility offers a different picture of urban segregation.

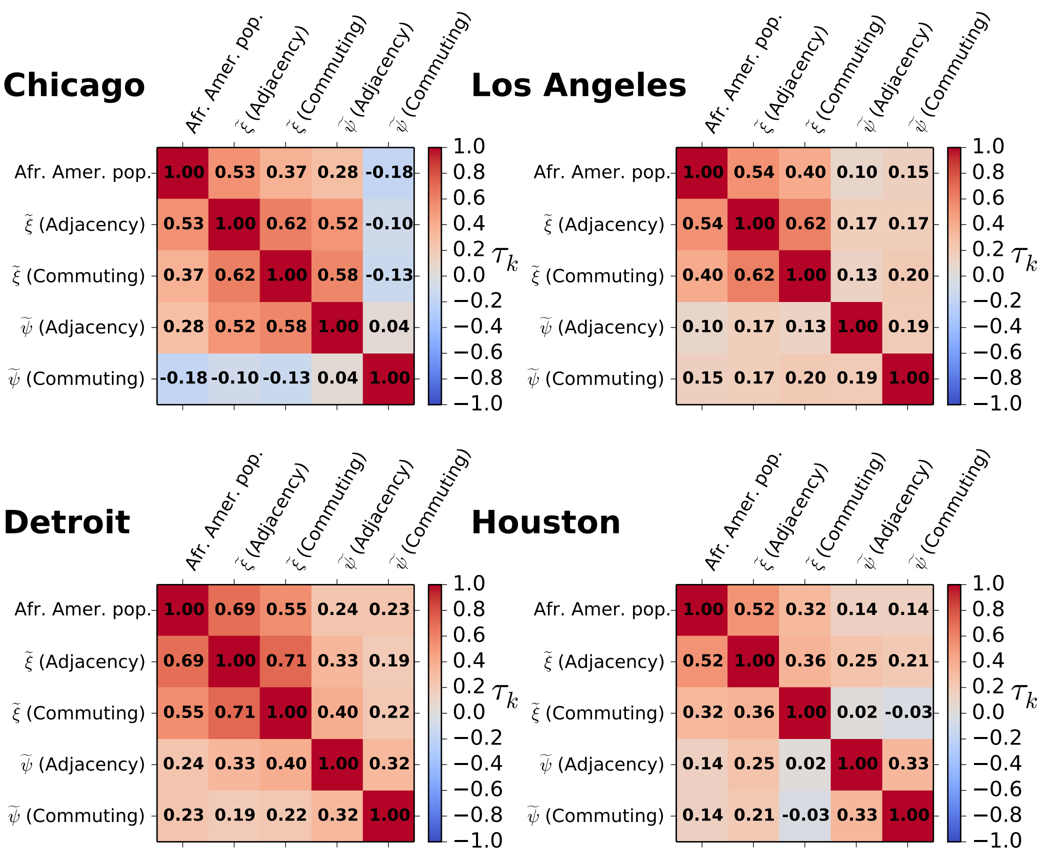

Fig. S5: **Correlations between the each of the local metrics of segregation $\tilde{\xi}$ and $\tilde{\psi}$ and the local ratio of African American population.** Correlation between each of the local indices of segregation and the ratio of African American population by census tract as well. On the top row Chicago and Los Angeles and on the bottom row Detroit and Houston.

Section S3: Temporal analysis of correlations with segregation indices and other socioeconomic indicators

In this section, we provide the temporal evolution of correlations between the difference in the percentage of COVID-19 incidence among African Americans and other segregation indices.

Statistical analysis of the COVID-19 incidence data

We provide in Fig. S6 the evolution of the difference in percentage of infected and deceased African Americans. States are split in quartiles of the distribution of the percentage of African Americans among the overall population. Regarding the difference on infected, while the average seems almost stable in most of the quartiles this is more a product of compensating changes than of stability in the values for a single state. For instance, in the first quartile there is a sharp increase in Minnesota compensated by a decrease in DC. On the third quartile, the sharp decrease in Illinois is compensated by the increase in Arkansas. It is also important to note that some states display strong discrepancies between the percentage on deceased and infected as, for instance, Minnesota.

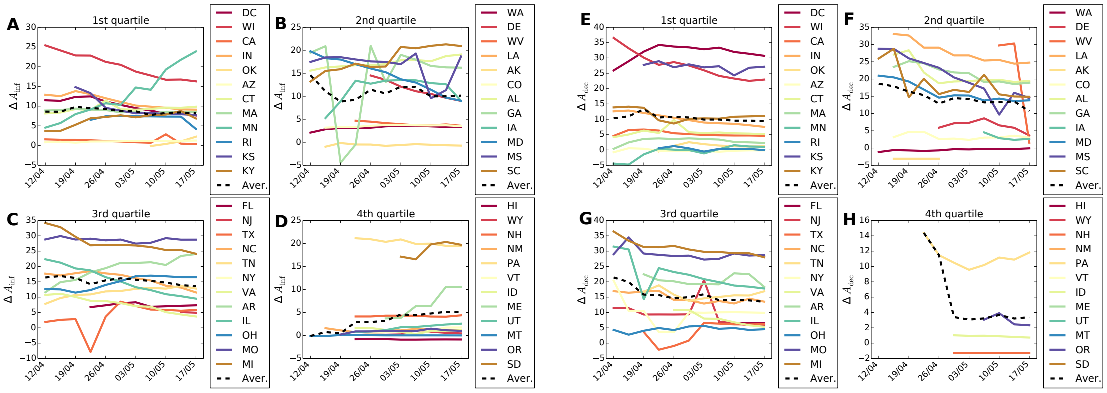

Fig. S6: The temporal evolution of the difference in percentage of **A-D** infected and **E-H** deceased African Americans by state. Each plot represents a quartile of the distribution of percentage of African American population.

Correlations with the difference in percentage of deceased African Americans

In the main manuscript, the main variable analysed is the difference in the percentage of African Americans since other factors might influence the deceased. In Fig. S7, we show the correlation with the difference in percentage of deceased African Americans. We can see that despite correlations are lower, they are stable across time. It is important to note that in the case of deceased individuals other factors like the age or the underlying health conditions might play a significant role. Again C and E computed in the commuting graph seem to outperform the rest of metrics.

Temporal evolution of correlations with another data set

We also had access to another project that aggregates data on the ethnicity of both infected and deceased African Americans by COVID-19 through three different temporal snapshots 22/04/2020, 04/05/2020 and 15/05/2020 [2]. In Fig. S8, we report the correlations in each of the three snapshots for the difference in the percentage of infected African Americans, which are in line with the results obtained for the other data set. Correlations are considerably high and significant for the first stages of the pandemic and decrease with time as the different lock-downs take place. As in the results provided in the main manuscript, those indices computed on the commuting graph seem to provide a better correlation than those computed on the adjacency one. Again those indices connected to the exposure of African Americans only yield significant correlation on the commuting network.

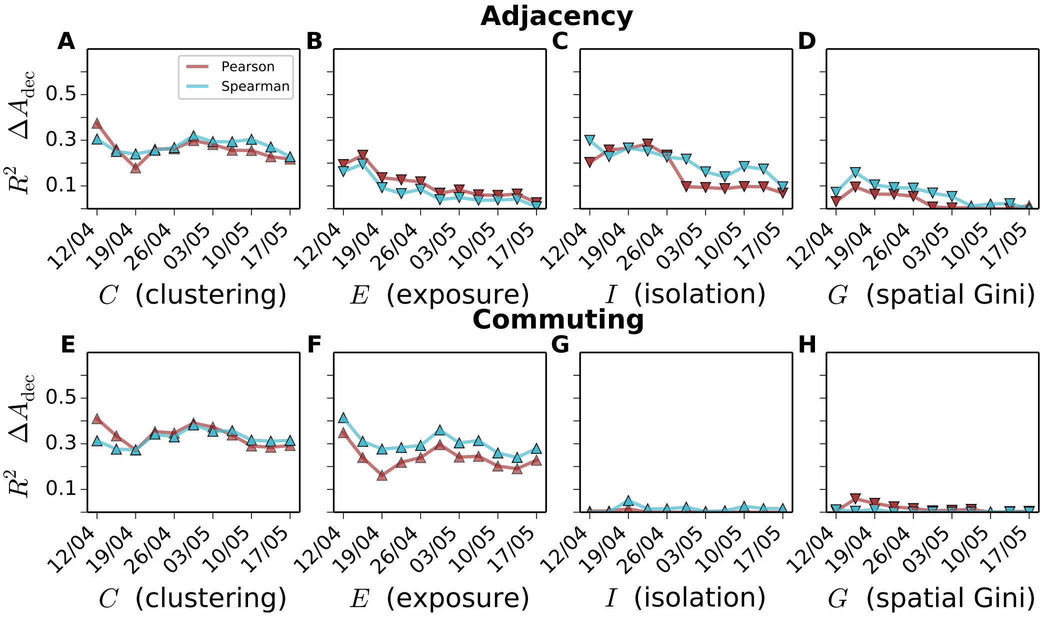

Fig. S7: Evolution of the Pearson and Spearman correlation (R^2) found between the difference in percentage of deceased African Americans and the four indices studied in the main manuscript. **A-D** Indices computed over the adjacency network:**A** C (clustering), **B** E (exposure), **C** I (isolation), **D** G (spatial Gini). **E-H** Indices computed over the commuting network: **E** C (clustering), **F** E (exposure), **G** I (isolation), **H** G (spatial Gini).

Formulation of the alternative indices C' and E'

In the main manuscript we have studied the metrics C and E which are computed from the elements of the normalised CMFPT $\widetilde{\tau}_{\alpha,\beta}$. However, there are more potential ways to capture the clustering and exposure of an ethnicity by doing the other calculations from that matrix. Here we propose the two alternative formulations for those two metrics

$$C' = \frac{\overline{\tau}_{OA}}{\overline{\tau}_{OO}}$$
$$E' = \frac{\overline{\tau}_{AA}}{\overline{\tau}_{AO}}$$

The first quantity comes from the ratio between the time from other ethnicities to African Americans and the time from other ethnicities to others, where higher values correspond more isolated African Americans compared to other ethnicities. The second quantity instead, is the ratio between the time separating African Americans and the time between African Americans and any other ethnicity, where higher values correspond to African Americans more exposed to others than to themselves. The correlation between our alternative proposals and the difference in the percentage of African Americans infected is shown in Fig. S9. While correlations are slightly lower, they are still significant. One interesting finding is that E' changes the sign of the correlation when computed over the adjacency graph and the commuting network. Highlighting once again the need of considering mobility to understand the segregation and exposure of ethnicities in urbanscapes.

Temporal evolution of correlations with other segregation indices from the literature

We have also studied the correlations between the difference in the percentage of COVID-19 incidence among African Americans and other segregation indices from the literature. First of all, we obtained the segregation index $\bar{\sigma}_\alpha$ proposed in [3], which is also based on the movement of random walks is spatial systems and captures the probability that a randomly chosen individual of group α meets another individual of the same group, or in this case, ethnicity. Additionally, we computed Moran's I, which is a measure of spatial auto-correlation and compares the ethnic composition of neighbourhoods [4]. The

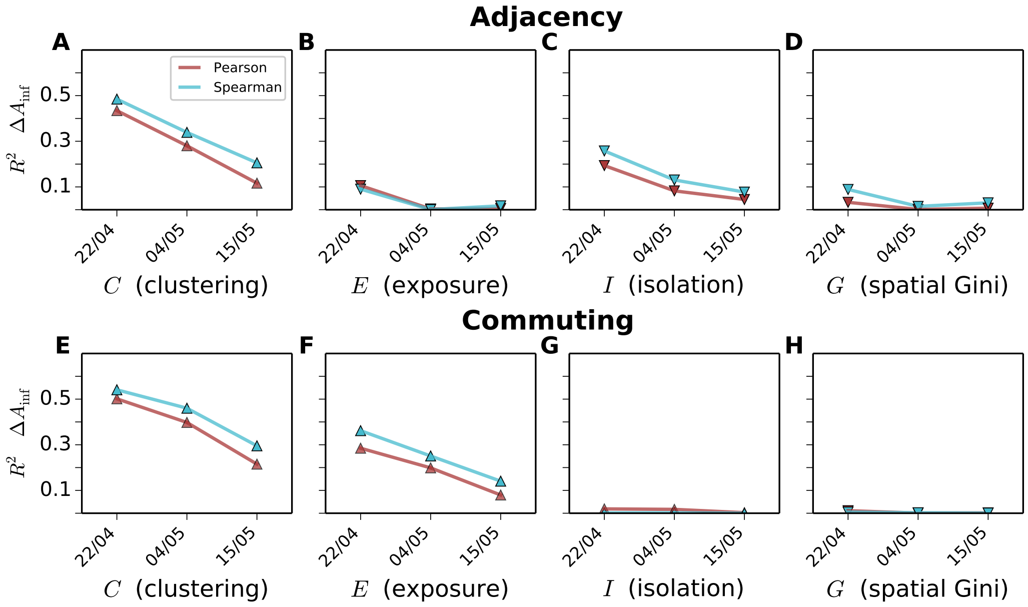

Fig. S8: Evolution of the Pearson and Spearman correlation (R^2) found between the difference on the infected African Americans and the four indices studied in the main manuscript using another data source. **A-D** Indices computed over the adjacency network:**A** C (clustering), **B** E (exposure), **C** I (isolation), **D** G (spatial Gini). **E-H** Indices computed over the commuting network: **E** C (clustering), **F** E (exposure), **G** I (isolation), **H** G (spatial Gini). The markers indicate the sign of the relation, positive for triangles pointing up and negative for triangles pointing down.

correlation of both metrics with the difference in the percentage of infected among African Americans. The evolution of the correlations is shown in Fig. S10, where only the Moran index calculated over the adjacency graph seems to display a significant correlation.

We considered a matrix of distance between ethnicities similar to the one obtained for $\widetilde{\tau}_{\alpha,\beta}$ using a measure proposed by [5]. Inspired by the Getis and Ord statistic [6], the metric proposed [5] quantifies for each location i the exposure of ethnicity α to ethnicity β as

$$ {}_{\alpha}^{\beta}G_i^* = \frac{\sum_{j=1}^{n} w_{ij}(\hat{d}_{pi}) m_{j,\beta}}{\sum_{j=1}^{n} m_{j,\beta}}, \tag{3} $$

where n is the total number of location in a city, j corresponds to each of those locations and $m_{j,\beta}$ is the population of ethnicity β in location j, $\hat{d}_{pi}$ is an estimate of trip length and $w_{ij}(\hat{d}_{pi})$ is a function of the distance that is equal to 1 when $d_{ij} < d_{pj}$ and 0 otherwise. In the case of the adjacency graph only adjacent pair of tracts were considered whereas in the case of the commuting network only pairs connected by commuting trips were considered. Overall ${}_{\alpha}^{\beta}G_i^*$ quantify the ratio of population of ethnicity β to which the individuals residing in i are exposed. In our case we set the threshold d_{pj} equal to the average commuting distance in each of the cities. Succinctly, ${}_{\alpha}^{\beta}G_i^*$ is a value between 0 and 1 that encapsulates the fraction of the population of ethnicity. We average the value of ${}_{\alpha}^{\beta}G_i^*$ to obtain a distance matrix between ethnicities in each of the cities as:

$$ {}_{\alpha}^{\beta}G_i^* = \sum_{i=1}^{n} m_{i,\alpha} {}_{\alpha}^{\beta}G_i^* \sum_{i=1}^{n} m_{i,\alpha}, \tag{4} $$

so that we take into account the fraction of population of ethnicity α in location i.

Finally from the matrix ${}_{\alpha}^{\beta} < G^* >$ we compute the same exposure and clustering indices computed

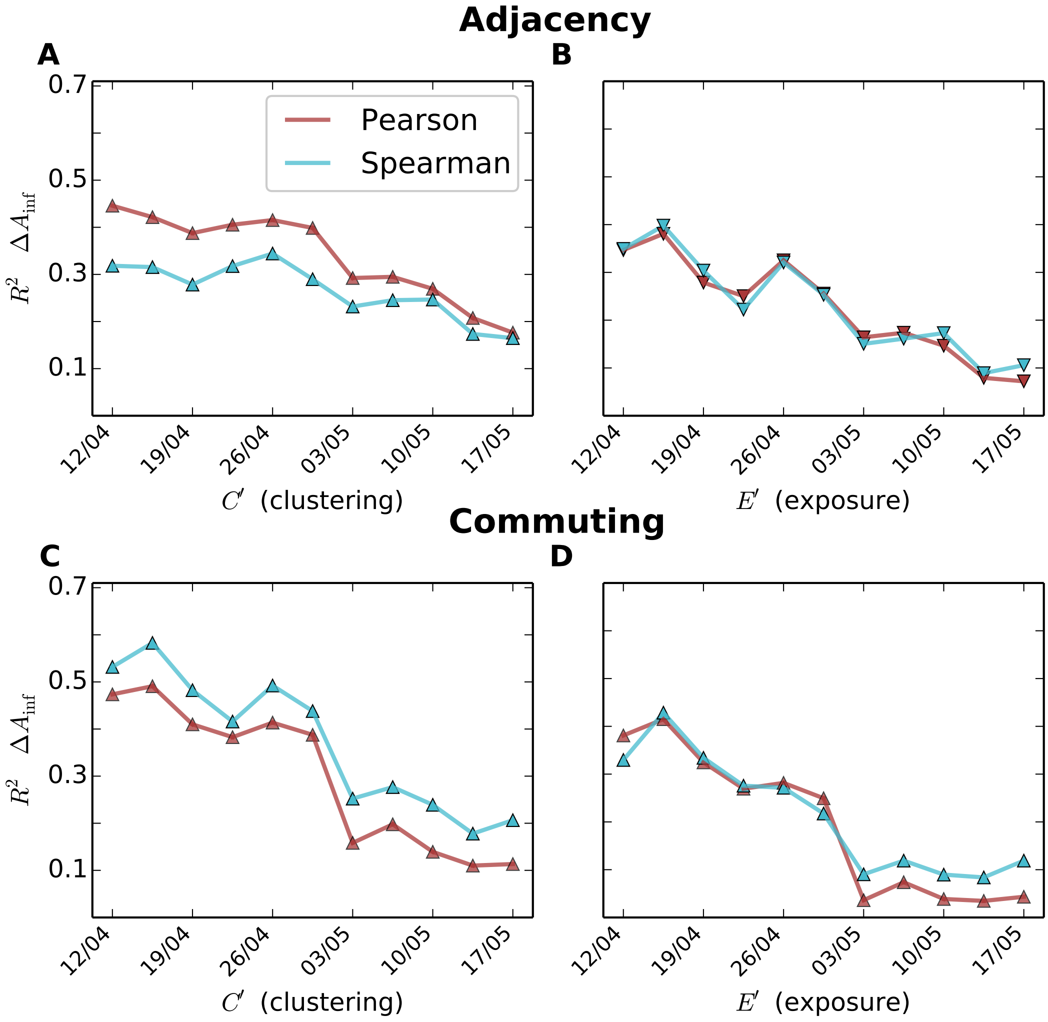

Fig. S9: Evolution of the Pearson and Spearman correlation (R^2) found between the difference of infected African Americans and the alternative indices proposed C' and E'. **A** C' (clustering) and **B** E' (exposure) calculated upon the adjacency network. **C** C' (clustering) and **D** E' (exposure) calculated upon the commuting network. The markers indicate the sign of the relation, positive for triangles pointing up and negative for triangles pointing down.

from $\widetilde{\tau}_{\alpha,\beta}$ in the main text. Calculating first

$$\overline{<G^*>}_{AO} = \frac{\sum_{\forall\beta\neq A} M^{\beta A}_{\alpha} <G^*>}{\sum_{\forall\beta\neq A} e^{\beta}}$$

$$\overline{<G^*>}_{OA} = \frac{\sum_{\forall\beta\neq A} M^{\beta\beta}_{A} <G^*>}{\sum_{\forall\beta\neq A} M^{\beta}}$$

$$\overline{<G^*>}_{OO} = \frac{\sum_{\forall\alpha,\beta\neq A} {}^{\beta}_{\alpha} <G^*> M^{\alpha} M^{\beta}}{\sum_{\forall\alpha,\beta\neq A} M^{\alpha} M^{\beta}},$$

to finally obtain

$$C^f = \frac{\overline{<G^*>}_{AO}}{\overline{<G^*>}_{OO}},$$

$$E^f = \frac{\overline{<G^*>}_{OO}}{\overline{<G^*>}_{OO}}.$$

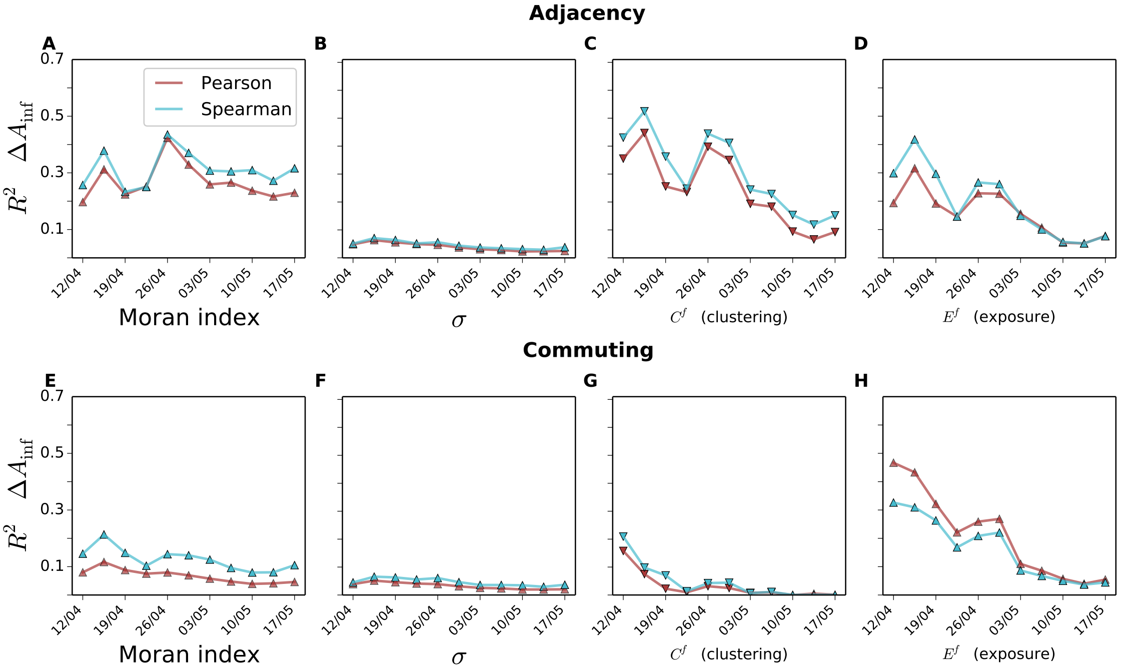

Fig. S10: **The temporal evolution of the correlation (R^2) between the incidence of COVID-19 in African American population and traditional segregation indices.** **A** σ and **B** Moran's I computed over the adjacency graph. **C,D** Correlation with the clustering C^f and exposure E^f indices computed over the adjacency graph. **E** σ and **F** Moran's I computed over the commuting graph. **G,H** Correlation with the clustering C^f and exposure E^f indices computed over the commuting network. The markers indicate the sign of the relation, positive for triangles pointing up and negative for triangles.

In Addition to the calculation of the indices in the adjacency and the commuting network with dynamical population, we computed the measures considering the residential population and the commuting network to investigate the role of the dynamical population. As can be seen in Fig. S10, significant correlations appear with all indices yet the higher values are with the exposure index especially when computed on the commuting network with dynamical population. Correlations are, however, lower and less stable than those obtained in the main manuscript. We provide in Table S2 the correlation obtained between the 10 classical and more recent segregation indicators we have analyzed through this work and the COVID-19 death gap suffered by African Americans. As for the case of the infection gap, the Perimeter/area ratio Spatial Dissimilarity as well as those measures based on [5] display a significant correlation for most of the dates we have analysed. The distance decay exposure and isolation also display a certain level of correlation for a few dates.

Overall, it is important to note that none of the additional segregation metrics we have studied in this section is more informative than the ones we proposed on the main manuscript based on CMFPT and CCT. Moreover, the use of the dynamical population together with the commuting network seems to improve some of the indices.

Date	Network	12/04	15/04	19/04	22/04	26/04	29/04	03/05	06/05	10/05	13/05	17/05
Bound. Spat. Dissim. [7]	Adj.	0.01	0.01	0.01	0.00	0.00	0.01	0.00	0.00	0.02	0.02	0.01
Bound. Spat. Dissim. [7]	Com.	0.02	0.00	0.00	0.00	0.00	0.01	0.00	0.00	0.02	0.01	0.00
Spat. Dissim. [7]	Adj.	0.09	0.22 *	0.18	0.16	0.14	0.10 *	0.11 **	0.11 *	0.06	0.07	0.08 *
Spat. Dissim. [7]	Com.	0.02	0.11	0.11	0.08	0.06	0.05	0.06	0.06	0.02	0.04	0.04
Per./area ratio Spat. Diss. [7]	Adj.	0.16 *	0.05	0.06	0.07 *	0.08 *	0.15	0.13	0.14	0.19	0.17	0.14
Per./area ratio Spat. Diss. [7]	Com.	0.34 *	0.45 **	0.40 **	0.36 ***	0.31 ***	0.35 ***	0.36 ***	0.36 ***	0.30 **	0.32 ***	0.30 ***
Dist. Decay Exposure [7]	Adj.	0.22	0.33 *	0.30	0.27	0.24 **	0.25 **	0.25 *	0.24 **	0.18	0.23	0.17 *
Dist. Decay Exposure [7]	Com.	0.17	0.28 *	0.26	0.23	0.20 **	0.19 *	0.19 *	0.19 **	0.10	0.14	0.14 *
Dist. Decay Isolation [7]	Adj.	0.30 *	0.41 **	0.36	0.32 *	0.27 ***	0.31 **	0.32 **	0.31 **	0.25 *	0.30	0.21 *
Dist. Decay Isolation [7]	Com.	0.21	0.34 *	0.30	0.27 *	0.22 **	0.23 **	0.24 **	0.23 **	0.14	0.18	0.18 *
$\bar{\sigma}_g$ [3]	Adj.	0.00	0.03	0.02	0.00	0.00	0.00	0.00	0.00	0.00	0.00	0.00
$\bar{\sigma}_g$ [3]	Com.	0.32 *	0.27	0.11	0.20	0.22	0.21	0.23	0.19	0.13	0.13	0.14
E^f [5]	Adj.	0.49 **	0.56 ***	0.46 **	0.40 **	0.35 ***	0.41 ***	0.44 **	0.39 **	0.30	0.31	0.33
E^f [5]	Com.	0.36	0.29	0.09	0.13	0.09	0.14	0.17	0.16	0.11	0.12	0.10
C^f [5]	Adj.	0.39 *	0.53 **	0.43 *	0.31 *	0.29 **	0.32 **	0.30 *	0.31 *	0.25	0.27	0.27
C^f [5]	Com.	0.27 ***	0.32 ***	0.26 **	0.25 **	0.29 **	0.29 **	0.28	0.26	0.25	0.24	0.23
Spatial Gini [7]	Adj.	0.03	0.10	0.06	0.06	0.05	0.01	0.00	0.00	0.00	0.00	0.01
Spatial Gini [7]	Com.	0.00	0.06	0.04	0.02	0.02	0.01	0.01	0.01	0.00	0.00	0.00
Moran's I [3]	Adj.	0.00	0.06	0.04	0.02	0.02	0.01	0.01	0.01	0.00	0.00	0.00
Moran's I [3]	Com.	0.00	0.06	0.04	0.02	0.02	0.01	0.01	0.01	0.00	0.00	0.00

Table S2: Table of correlations for the death gap of African Americans (Pearson R^2) obtained with additional widely used segregation indicators. All the indices are obtained by comparing (ratio) the segregation of African Americans with the average of the other ethnicities. Except from C^f, E^f, $\bar{\sigma}_g$ and Spatial Gini in the commute graph, all indicators were calculated using the PySAL package in Python [7].

Section S4: The relation between the COVID-19 infection gap in African Americans and socio-economic indicators

We present in this section the correlations between a set of socio-economic indicators and the incidence of COVID-19 in African Americans. For the sake of brevity, we focus here only on the data set used in the main manuscript as well as in the difference in the percentage of infections which is the case where correlations are higher. The set of indicators we have studied are the median household income, the percentage of the population below the poverty level, the percentage of insured and uninsured African Americans, the usage of public transportation by both African Americans and the overall population, the percentage of African American population in a state, the average commuting distance and the ratio between the average commuting distance of African Americans and the overall population. All of the metrics are provided at the level of the African American population and the results are shown in Fig. S11. The median household income, the percentage of the population below the poverty level, the percentage of insured and uninsured African Americans were obtained from the 2018 American Community Survey elaborated by the U.S. Census Bureau [9]. Most of the variables yield low or very low correlations except for the usage of public transportation by African Americans. Economic indicators such as median income or percentage of poverty seem to slightly correlate with the incidence of COVID-19, which could because because a more deprived African American community puts them in a more risky situation. Regarding the health indicators related to the degree of insurance of African Americans, it seems there is no direct relation with the number of infected. Not so surprising results since we are analysing the percentage of infected and, therefore, the fact of having insurance might not change significantly the risk of getting the illness. Finally, the usage of public transportation seems to play a crucial role in the spread of the disease, especially if we compare the use done by the African American population and the overall population where no correlation appears. The fact that African Americans use more public transportation might put them on a more dangerous position as well as might a reflection of their economic status. Moreover, it could happen that in those cities in which African Americans are more segregated they also have to use more the public transportation.

Additionally to those socio-economic variables we also tested if the overall African American population can also be used as a proxy for the difference in percentage. We also computed on our commuting networks the average commuting distance of the African American population as well as the ratio with the commuting distance of the overall population. As displayed in Fig. S11, the overall percentage of African American population seems to be related to the difference in the percentage of infected. However, there is a striking difference between the Pearson and the Spearman correlation coefficients, which means that the rank is more or less conserved yet there are strong outliers. In other words, a state with more percentage of African American population will more easily have a higher difference on the infected yet the population does not align the points in a straight trend. Regarding the mobility indicators, none of them yields a significant correlation, meaning that it is not so relevant how far African Americans travel and where they travel and whom they meet.

Even though the single variable analysis points out that socioeconomic indicators are less informative to understand the infection gap than our segregation measures, we have checked for possible confounding factors by performing a multivariate analysis. For each of the socioeconomic indicators, we have performed an analysis of variance when the clustering (G) and exposure (E) are computed in the adjacency graph. The results are summarized in Fig. S12 for the infection gap by the 12th of April 2020 and in Fig. S13 for the death gap by the 12th of April 2020. For all socio-economic indicators, our clustering and exposure indicators explain more variance on the infection gap of African Americans than the secondary variable for both dates. However, two of them display a significant correlation: the median household income and the life expectancy. Each of them captures disparities suffered by African Americans in a different aspect. The median income is an indicator of the economic status of African Americans and could be also interpreted as a proxy for better work conditions or less face-to-face jobs. In the case of life expectancy, it can be understood as a proxy for worth health conditions, which would explain the significant correlations. Socio-economic indicators also provide high correlation in the case of the death gap.

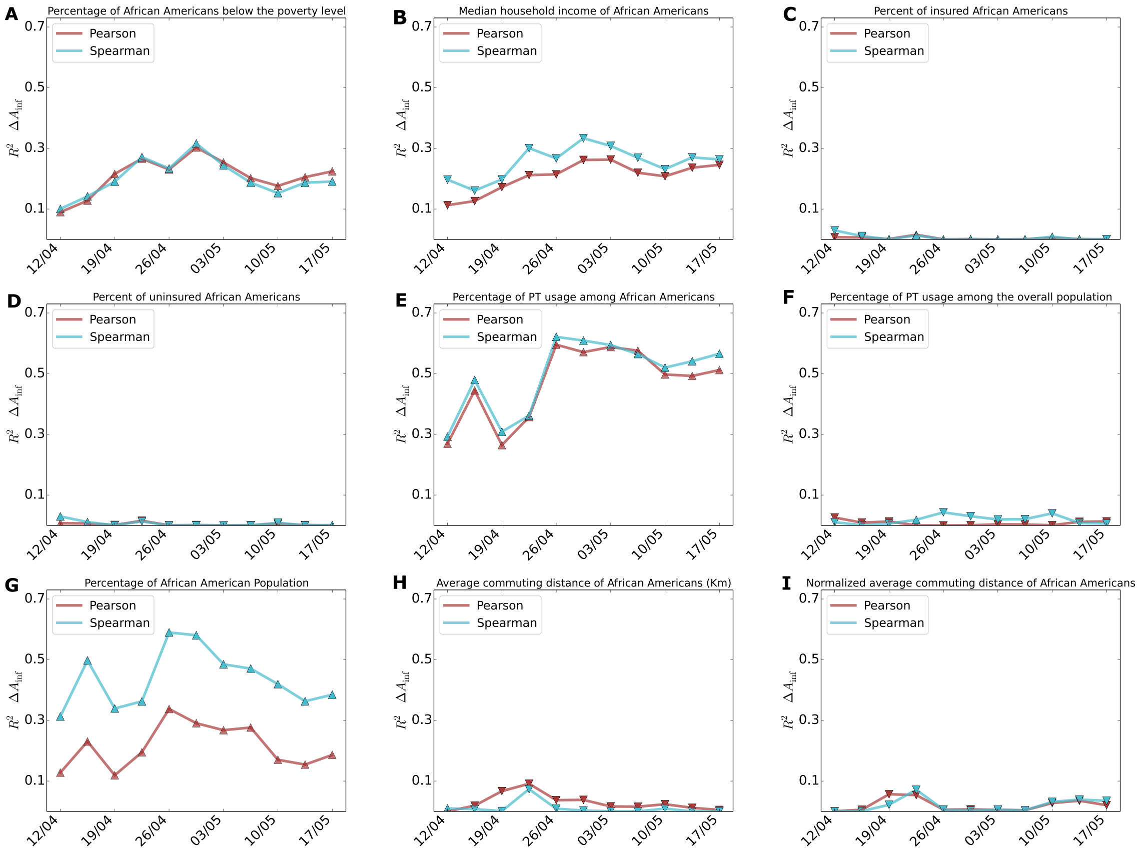

Fig. S11: **The temporal evolution of Pearson and Spearman correlations (R^2) between the incidence of COVID-19 in African American population and a set of socio-economic and mobility indicators.** On the top row have **A** the median household income of African Americans, **B** the percentage of African Americans below the poverty level and **C** the percent of insured African Americans. On the middle row there is **D** the percent of uninsured African Americans, **E** the percentage of use of public transportation among African Americans and **F** the percentage of use among the overall population. On the bottom row **G** the percentage of African Americans among the population, **H** the average commuting distance of African Americans and **I** its ratio with the commuting distance of the overall population. The markers indicate the sign of the relation, positive for triangles pointing up and negative for triangles.

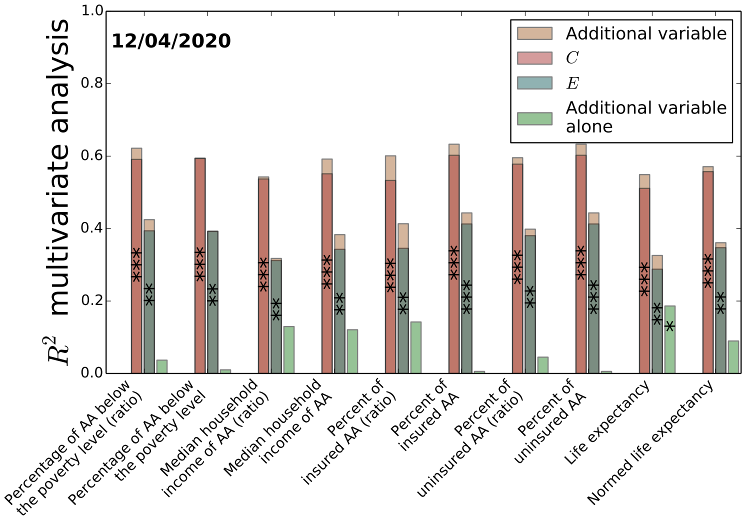

Fig. S12: **Multivariate analysis for the incidence of COVID-19 in African American population as a function of a set of socio-economic indicators and the clustering (G) and exposure (E) by the 12th of April 2020.** For each socio-economic indicator, we display the variance explained by the additional variable in yellow and the clustering(G) in red and the total, which corresponds to the sum of both bars. Similarly, we display the variance explained by the additional variable in yellow and the exposure (G) in dark green and the total, which corresponds to the sum of both bars. Finally the light green bar corresponds to the single variable analysis performed only with the socio-economic indicator.

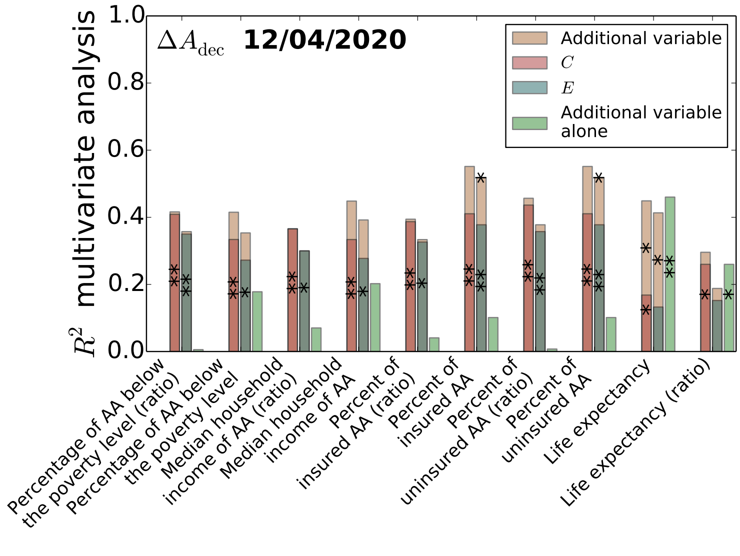

Fig. S13: **Multivariate analysis for the incidence of COVID-19 in African American population as a function of a set of socio-economic indicators and the clustering (G) and exposure (E) by the 12th of April 2020.** For each socio-economic indicator, we display the variance explained by the additional variable in yellow and the clustering(G) in red and the total, which corresponds to the sum of both bars. Similarly, we display the variance explained by the additional variable in yellow and the exposure (G) in dark green and the total , which corresponds to the sum of both bars. Finally the light green bar corresponds to the single variable analysis performed only with the socio-economic indicator

Supplementary References

[1] Black Population in US | BlackDemographics.com; 2016. [Online; accessed 2020-05-30]. https://blackdemographics.com/black-covid-19-tracker/.

[2] COVID Racial Data Tracker; 2016. [Online; accessed 2020-05-30]. https://covidtracking.com/race.

[3] Ballester C, Vorsatz M. Random walk-based segregation measures. Review of Economics and Statistics. 2014;96(3):383–401.

[4] Cliff AD, Ord JK. Spatial processes: models & applications. Taylor & Francis; 1981.

[5] Farber S, Páez A, Morency C. Activity spaces and the measurement of clustering and exposure: A case study of linguistic groups in Montreal. Environment and Planning A. 2012;44(2):315–332.

[6] Getis A, Ord JK. The analysis of spatial association by use of distance statistics. In: Perspectives on spatial data analysis. Springer; 2010. p. 127–145.

[7] Rey SJ, Anselin L. PySAL: A Python library of spatial analytical methods. In: Handbook of applied spatial analysis. Springer; 2010. p. 175–193.

[8] Rey SJ, Anselin L. PySAL: A Python Library of Spatial Analytical Methods. The Review of Regional Studies. 2007;37(1):5–27.

[9] Manson S, Schroeder J, Riper DV, Ruggles S. IPUMS National Historical Geographic Information System: Version 14.0 [Database]. Minneapolis, MN: IPUMS. 2019.;. http://doi.org/10.18128/D050.V14.0.
